# Follow-up strategies for patients with splenic trauma managed non-operatively: the 2022 World Society of Emergency Surgery consensus document

**DOI:** 10.1186/s13017-022-00457-5

**Published:** 2022-10-12

**Authors:** Mauro Podda, Belinda De Simone, Marco Ceresoli, Francesco Virdis, Francesco Favi, Johannes Wiik Larsen, Federico Coccolini, Massimo Sartelli, Nikolaos Pararas, Solomon Gurmu Beka, Luigi Bonavina, Raffaele Bova, Adolfo Pisanu, Fikri Abu-Zidan, Zsolt Balogh, Osvaldo Chiara, Imtiaz Wani, Philip Stahel, Salomone Di Saverio, Thomas Scalea, Kjetil Soreide, Boris Sakakushev, Francesco Amico, Costanza Martino, Andreas Hecker, Nicola de’Angelis, Mircea Chirica, Joseph Galante, Andrew Kirkpatrick, Emmanouil Pikoulis, Yoram Kluger, Denis Bensard, Luca Ansaloni, Gustavo Fraga, Ian Civil, Giovanni Domenico Tebala, Isidoro Di Carlo, Yunfeng Cui, Raul Coimbra, Vanni Agnoletti, Ibrahima Sall, Edward Tan, Edoardo Picetti, Andrey Litvin, Dimitrios Damaskos, Kenji Inaba, Jeffrey Leung, Ronald Maier, Walt Biffl, Ari Leppaniemi, Ernest Moore, Kurinchi Gurusamy, Fausto Catena

**Affiliations:** 1 Department of Surgical Science, Emergency Surgery Unit, University of Cagliari, Cagliari, Italy; 2 Department of Emergency, Digestive and Metabolic Minimally Invasive Surgery, Poissy and Saint Germain en Laye Hospitals, Poissy, France; 3 General and Emergency Surgery Department, School of Medicine and Surgery, Milano-Bicocca University, Monza, Italy; 4 Trauma and Acute Care Surgery Department, Niguarda Hospital, Milan, Italy; 5 Department of Emergency and Trauma Surgey, Bufalini Trauma Center, Cesena, Italy; 6 Department of Gastrointestinal Surgery, Stavanger University Hospital University of Bergen, Stavanger, Norway; 7 General, Emergency and Trauma Surgery Department, Pisa University Hospital, Pisa, Italy; 8 Department of Surgery, Macerata Hospital, Macerata, Italy; 9 Department of General Surgery, Dr Sulaiman Al Habib/Alfaisal University, Riyadh, Saudi Arabia; 10 School of Medicine and Health Science, University of Otago, Wellington Campus, Wellington, New Zealand; 11 Division of General Surgery, IRCCS Policlinico San Donato, University of Milan, Milan, Italy; 12 Department of Applied Statistics, The Research Office, College of Medicine and Health Sciences United Arab Emirates University, Abu Dhabi, UAE; 13 Department of Traumatology, John Hunter Hospital and University of Newcastle, Newcastle, NSW Australia; 14 Government Gousia Hospital, Srinagar, India; 15 Department of Surgery, East Carolina University, Brody School of Medicine, Greenville, USA; 16 Department of Surgery, San Benedetto del Tronto Hospital, AV5, San Benedetto del Tronto, Italy; 17 Shock Trauma Center, University of Maryland School of Medicine, Baltimore, USA; 18 Research Institute of Medical University Plovdiv/University Hospital St George Plovdiv, Plovdiv, Bulgaria; 19 Trauma Service, John Hunter Hospital, Newcastle, Australia; 20 The University of Newcastle, Newcastle, Australia; 21 Department of Anesthesiology and Acute Care, Umberto I Hospital of Lugo, Ausl della Romagna, Lugo, Italy; 22 Department of General and Thoracic Surgery, University Hospital of Giessen, Giessen, Germany; 23 Unit of General Surgery, Henri Mondor Hospital, UPEC, Créteil, France; 24 Service de Chirurgie Digestive, Centre Hospitalier Universitaire Grenoble Alpes, Grenoble, France; 25 Trauma Department, University of California, Davis, Sacramento, CA USA; 26 General, Acute Care and Trauma Surgery Foothills Medical Centre, University of Calgary, Calgary, AB Canada; 27 General Surgery, Hospital, National and Kapodistrian University of Athens (NKUA), Athens, Greece; 28 Division of General Surgery, Rambam Health Care Campus, Haifa, Israel; 29 Department of Surgery, Denver Health Medical Center, Denver, CO USA; 30 Unit of General Surgery, San Matteo Hospital, Pavia, Italy; 31 Division of Trauma Surgery, University of Campinas, Campinas, SP Brazil; 32 Director of Trauma Services, Auckland City Hospital, Auckland, New Zealand; 33 UOC Chirurgia Digestiva e d’Urgenza, Azienda Ospedaliera S.Maria, Terni, Italy; 34 Department of Surgical Sciences and Advanced Technologies, University of Catania, Catania, Italy; 35 Department of Surgery, Tianjin Nankai Hospital, Nankai Clinical School of Medicine, Tianjin Medical University, Tianjin, China; 36 Riverside University Health System Medical Center, Moreno Valley, CA USA; 37 Anesthesia and Intensive Care Unit, AUSL Romagna, M. Bufalini Hospital, Cesena, Italy; 38 Department of General Surgery, Military Teaching Hospital, Hôpital Principal Dakar, Dakar, Senegal; 39 Department of Surgery, Radboudumc, Nijmegen, The Netherlands; 40 Department of Anesthesia and Intensive Care, Parma University Hospital, Parma, Italy; 41 Department of Surgical Disciplines, Immanuel Kant Baltic Federal University, Regional Clinical Hospital, Kaliningrad, Russia; 42 Department of General Surgery, Royal Infirmary Edinburgh, Edinburgh, UK; 43 University of Southern California, Los Angeles, USA; 44 Division of Surgery and Interventional Science, University College London (UCL), London, UK; 45 Milton Keynes University Hospital, Milton Keynes, UK; 46 University of Washington, Seattle, USA; 47 Division of Trauma and Acute Care Surgery, Scripps Clinic Medical Group, La Jolla, La Jolla, CA USA; 48 Abdominal Center, Helsinki University Hospital and University of Helsinki, Helsinki, Finland; 49 Ernest E. Moore Shock Trauma Center, University of Colorado School of Medicine, Denver, CO USA

**Keywords:** Spleen, Trauma, Nonoperative management, Conservative treatment, Diagnostic imaging, Follow-up, Embolization, Consensus

## Abstract

**Background:**

In 2017, the World Society of Emergency Surgery published its guidelines for the management of adult and pediatric patients with splenic trauma. Several issues regarding the follow-up of patients with splenic injuries treated with NOM remained unsolved.

**Methods:**

Using a modified Delphi method, we sought to explore ongoing areas of controversy in the NOM of splenic trauma and reach a consensus among a group of 48 international experts from five continents (Africa, Europe, Asia, Oceania, America) concerning optimal follow-up strategies in patients with splenic injuries treated with NOM.

**Results:**

Consensus was reached on eleven clinical research questions and 28 recommendations with an agreement rate ≥ 80%. Mobilization after 24 h in low-grade splenic trauma patients (WSES Class I, AAST Grades I–II) was suggested, while in patients with high-grade splenic injuries (WSES Classes II–III, AAST Grades III–V), if no other contraindications to early mobilization exist, safe mobilization of the patient when three successive hemoglobins 8 h apart after the first are within 10% of each other was considered safe according to the panel. The panel suggests adult patients to be admitted to hospital for 1 day (for low-grade splenic injuries—WSES Class I, AAST Grades I–II) to 3 days (for high-grade splenic injuries—WSES Classes II–III, AAST Grades III–V), with those with high-grade injuries requiring admission to a monitored setting. In the absence of specific complications, the panel suggests DVT and VTE prophylaxis with LMWH to be started within 48–72 h from hospital admission. The panel suggests splenic artery embolization (SAE) as the first-line intervention in patients with hemodynamic stability and arterial blush on CT scan, irrespective of injury grade. Regarding patients with WSES Class II blunt splenic injuries (AAST Grade III) without contrast extravasation, a low threshold for SAE has been suggested in the presence of risk factors for NOM failure. The panel also suggested angiography and eventual SAE in all hemodynamically stable adult patients with WSES Class III injuries (AAST Grades IV–V), even in the absence of CT blush, especially when concomitant surgery that requires change of position is needed. Follow-up imaging with contrast-enhanced ultrasound/CT scan in 48–72 h post-admission of trauma in splenic injuries WSES Class II (AAST Grade III) or higher treated with NOM was considered the best strategy for timely detection of vascular complications.

**Conclusion:**

This consensus document could help guide future prospective studies aiming at validating the suggested strategies through the implementation of prospective trauma databases and the subsequent production of internationally endorsed guidelines on the issue.

## Background

The spleen is the most commonly injured solid organ in blunt abdominal trauma. Over the last few decades, there has been a considerable shift toward a more conservative approach in the management of splenic trauma, with an emphasis on the preservation of splenic function [1] and, currently, Nonoperative Management (NOM) has become the standard management technique in hemodynamically stable patients with spleen injuries.

In western countries, 90–85% of traumatic splenic injury patients receive NOM, while the remainder has an immediate splenectomy for hemorrhagic shock or hemodynamic instability [2]. Current data suggest NOM has a success rate of up to 95% [1, 3–5], and the effectiveness of conservative treatment decreases as the AAST (American Association for the Surgery of Trauma) grade increases: NOM failure rate (without splenic artery embolization-SAE-) is reported to be 2–10% for AAST Grades I–II, 10–20% for AAST Grade III, 40–43% for AAST Grade IV, and up to 70–75 for AAST Grade V injuries [3, 6, 7].

NOM for splenic trauma usually consists of clinical observation, but may include the use of SAE. SAE has expanded the role of NOM, treating splenic artery injuries in case of active bleeding and non-bleeding injuries, such as pseudo-aneurysm (PSA) and arteriovenous fistula, as well as managing complications in stable patients [8, 9]. SAE in high-grade splenic injuries has been widely associated with a decrease in NOM failure and a lower incidence of splenectomy, with a splenic salvage rate of over 90% in all injury grades, and greater than 80% for the most severe injuries [6].

As splenic injuries can be fatal not only at the admission of the patient to the Emergency Department (ED) but also due to delayed splenic hematoma or PSA rupture, standardized recommendations in the follow-up of splenic trauma patients treated with NOM are necessary.

In 2017, the World Society of Emergency Surgery published its guidelines for the management of adult and pediatric patients with splenic trauma [1]. However, several issues regarding the follow-up of patients with splenic injury treated with NOM remained unsolved. The optimal patient selection for SAE, the need for routine follow-up imaging, the need for vaccinations after SAE, the recommended length of hospital stay, the correct timing of bed rest and resumption of activity, and the timing of institution of deep vein thrombosis (DVT) prophylaxis are still controversial [10–12].

Using a modified Delphi method, we sought to explore ongoing areas of controversy in the NOM of splenic trauma and reach a consensus among a group of 48 international experts from five continents (Africa, Europe, Asia, Oceania, America) concerning optimal follow-up strategies in patients with splenic injuries treated with NOM.

## Methods

This consensus document has been created by a WSES (World Society of Emergency Surgery) collaborative group to identify clinically relevant questions and encourage the members of the experts' panel to finally achieve a Consensus on the main topics of interest regarding the follow-up of patients with splenic trauma treated with NOM.

A modified Delphi method was implemented to reach the Consensus. The chairs of the Consensus (FC, MP) selected panel members based on contributions to the peer-reviewed literature on abdominal trauma. Forty-eight experts were identified based on this method, and a personal email invitation to participate in the Consensus was sent to them.

### Topics elaboration and prioritization

The subject of follow-up of splenic trauma patients treated with NOM was divided into 11 main research questions. Research topics and questions were formulated, revised, and approved by all experts in two subsequent Delphi rounds in February 2022–March 2022. Each panel member was sent a questionnaire with instructions to comment on each topic based on their personal opinion, experience, or previous research.

**Fig. 1:**
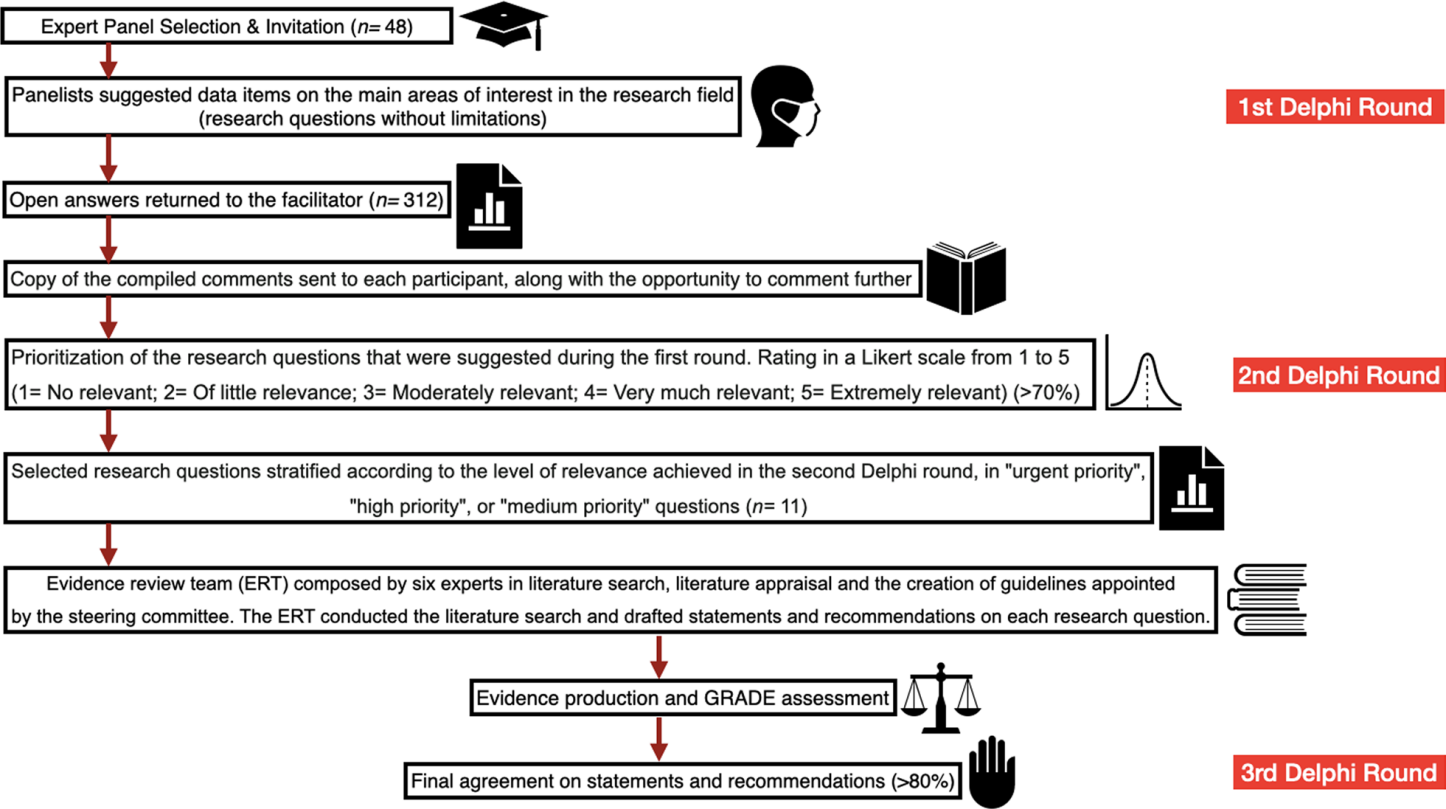
Summary of the modified Delphi process

After each Delphi round, the panel was presented with an aggregated summary of the previous round, allowing each expert to adjust their answers according to the group response. In the first Delphi round, the panel members were encouraged to suggest up to ten relevant research questions related to the follow-up of patients with splenic trauma treated with NOM. The first Delphi round was closed on Sunday March 6, 2022. Forty-two members of the experts' panel took part in the first round, and 312 open answers were returned to the facilitator, who grouped the comments and prepared copies of the information. A copy of the compiled comments was sent to each participant, along with the opportunity to comment further. The second round of the Delphi process involved the prioritization of the clinically relevant research questions that were suggested during the first round. The panel was called to rate on a Likert scale from 1 to 5 (1 = No relevant; 2 = Of little relevance; 3 = Moderately relevant; 4 = Very much relevant; 5 = Extremely relevant) the research questions to be explored in the literature review. The second Delphi round was closed on Sunday, March 20, 2022. The Consensus was defined as > 70% of scores ranging from 4 to 5. The selected research questions were finally stratified according to the level of relevance achieved in the second Delphi round, in "urgent priority," "high priority," or "medium priority" questions. The steering committee appointed an evidence review team (ERT) composed of six experts in the literature search, appraisal, and the creation of guidelines. The ERT conducted the literature search and drafted statements and recommendations on each research question (Fig. 1).

### Literature review

The literature review process was carried out conforming to the 2020 update of the PRISMA statement standards for systematic reviews and meta-analyses [13] between February 26, 2022, and April 22, 2022. MEDLINE (via PubMed), Google Scholar, the Cochrane Central Register of Controlled Trials, and EMBASE were systematically searched for relevant studies. Citations were included for the period between January 2000 and March 2022.

Studies were identified in PubMed using the MeSH terms "Splenic trauma" OR "Splenic injury" AND "Follow-up." Similar search strategies were performed in Embase and Google Scholar. The Cochrane database was searched for all articles relating to splenic trauma. After performing different searches, duplicates were identified and deleted. To increase the yield of the systematic search, the reference list of relevant articles and guidelines for treating patients with splenic trauma were searched.

Study inclusion criteria included systematic reviews with or without meta-analyses, randomized controlled trials, nonrandomized cohort studies (including registry studies), and case series on the follow-up of patients with splenic injuries treated with NOM, published in the English language between January 2000 and March 2022. Animal studies, case reports, narrative reviews, commentaries, and studies on splenic injuries not including specific information on the type of NOM were excluded. All relevant information was reported and discussed to answer the research questions.

### The GRADE methodology

The recommendations were formulated and graded according to the Grading of Recommendations Assessment, Development and Evaluation (GRADE) hierarchy of evidence [14], summarized in Table 1. The quality of evidence (QoE) was marked as high, moderate, low, or very low. This could be either downgraded in case of significant bias or upgraded when multiple high-quality studies showed consistent results. The highest quality of evidence studies (systematic reviews with meta-analysis of randomized controlled trials) was assessed first. If the meta-analyses were of sufficient quality, they were used to answer the research question. If no meta-analysis of sufficient quality was found, randomized controlled trials and nonrandomized cohort studies were evaluated. The strength of the recommendation (SoR) was based on the level of evidence and qualified as weak (defined as a suggestion or conditional recommendation) or strong (recommendation) [15, 16]. The panel group reviewed the content and strength of each statement and recommendation following the informative documents provided by the ERT during the third and last Delphi round.

**Table 1:** GRADE Quality of evidence and strength of recommendations

Quality of evidence and strength of recommendation	Clarity of balance between desirable and undesirable effects	Methodological quality of supporting evidence	Implications
High-quality evidence, strong recommendation	Desirable effects clearly outweigh undesirable effects, or vice versa	Consistent evidence from well-performed RCTs or exceptionally strong evidence from unbiased observational studies	Recommendation can apply to most patients in most circumstances. Further research is unlikely to change our confidence in the estimate effect
Moderate-quality evidence, strong recommendation	Desirable effects clearly outweigh undesirable effects, or vice versa	Evidence from RCTs with important limitations (inconsistent results, methodological flaws, indirectness, imprecision) or exceptionally strong evidence from unbiased observational studies	Recommendation can apply to most patients in most circumstances. Further research (if performed) is likely to have an important impact on our confidence in the estimate of effect and may change the estimate
Low-quality evidence, strong recommendation	Desirable effects clearly outweigh undesirable effects, or vice versa	Evidence for at least one critical outcome from observational studies, RCTs with serious flaws or indirect evidence	Recommendation may change when higher quality evidence becomes available. Further research (if performed) is likely to have an important impact on our confidence in the estimate of effect and is likely to change the estimate
Very low-quality evidence, strong recommendation (rarely applicable)	Desirable effects clearly outweigh undesirable effects, or vice versa	Evidence for at least one critical outcome from unsystematic clinical observations or very indirect evidence	Recommendation may change when higher quality evidence becomes available; any estimate of effect for at least one critical outcome is very uncertain
High-quality evidence, weak recommendation	Desirable effects closely balanced with undesirable effects	Consistent evidence from well-performed RCTs or exceptionally strong evidence from unbiased observational studies	The best action may differ depending on circumstances or patients or societal values. Further research is unlikely to change our confidence in the estimate effect
Moderate-quality evidence, weak recommendation	Desirable effects closely balanced with undesirable effects	Evidence from RCTs with important limitations (inconsistent results, methodological flaws, indirectness, imprecision) or exceptionally strong evidence from unbiased observational studies	Alternative approaches likely to be better for some patients under some circumstances. Further research (if performed) is likely to have an important impact on our confidence in the estimate of effect and may change the estimate
Low-quality evidence, weak recommendation	Uncertainty in the estimates of desirable effects, harms, and burden; desirable effects, harms, and burden may be closely balanced	Evidence for at least one critical outcome from observational studies, RCTs with serious flaws or indirect evidence	Other alternatives may be equally reasonable. Further research is very likely to have an important impact on our confidence in the estimate of effect an is likely to change the estimate
Very low-quality evidence, weak recommendation	Major uncertainty in the estimates of desirable effects, harms, and burden; desirable effects may or may not be balanced with undesirable effects	Evidence for at least one critical outcome from unsystematic clinical observations or very indirect evidence	Other alternatives may be equally reasonable. Any estimate of effect, for at least one critical outcome, is very uncertain

### Agreement on statements and recommendations

The Delphi methodology was implemented to reach an agreement among the experts on all statements and recommendations. Each was subject to voting by the experts' panel using the Google Forms online platform. When a unanimous consensus was not reached, supporting evidence from the systematic review of the literature performed for the specific research question was presented and discussed, and, if necessary, the second round of voting was carried out. Each recommendation was then voted upon by the panel, and consensus was considered to have been reached if at least 80% of votes were in favor of the statement. Where there was discordance, the recommendation was improved with panel input until approval was granted (Table 2).

**Table 2:** Summary of the 2022 WSES consensus on the follow-up strategies for patients with splenic trauma treated with non-operative management (NOM). Statements and recommendations

**Research Question: 1.** What is the optimal duration of bed-rest for patients treated with NOM for splenic trauma according to the injury grade?	
**Priority level:** *High*	
**Statement:** Abbreviated bed rest of 24 h is safe and does not increase NOM failure. The panel agrees that early mobilization could be considered in all splenic injury patients according to their general condition and associated injuries **[Quality of Evidence: Moderate]**. The day on which mobilization was performed is not associated with an increased risk of delayed hemorrhage in adult patients with low (WSES Class I, AAST Grades I–II) and high-grade (WSES Classes II–III, AAST Grades III–V) splenic injuries **[Quality of Evidence: Moderate]**. Current evidence supports an abbreviated period of bed rest of 24 h or less after splenic injury for hemodynamically stable children whose hemoglobin has been documented to be stable **[Quality of Evidence: Moderate]**	**Recommendation:** The panel suggests allowing early mobilization within 24 h in patients with low-grade (WSES Class I, AAST Grades I–II) splenic injuries treated with NOM. Patients with WSES Class II (AAST Grade III) splenic injuries can be mobilized after 2 days from the trauma if no other contraindications exist **[Strength of Recommendation: Conditional recommendation, based on Low quality of evidence, Agreement 89.1%]**. In patients with high-grade splenic injuries (WSES Class III, AAST Grades IV–V), if no other contraindications to early mobilization exist, the patient can be mobilized safely after 2 days from the trauma when three successive hemoglobins 8 h apart after the first are within 10% of each other, and if clinical parameters remain stable **[Strength of Recommendation: Conditional recommendation, based on Low quality of evidence, Agreement 87.2%]**. The panel suggests a shortened protocol of one night of bed rest for WSES Class I injuries (AAST Grade II; no need for bed rest for AAST Grade I) and two nights for WSES Classes II–III (AAST Grade ≥ III) in children when clinical parameters remain stable **[Strength of Recommendation: Conditional recommendation, based on Moderate quality of evidence, Agreement 82.6%]**
**Research Question: 2.** What is the optimal timing and type of anti-thrombotic prophylaxis for patients with splenic trauma treated with NOM?	
**Priority level:** *High*	
**Statement:** In the absence of contraindications related to associated injuries or conditions, such as intracranial hemorrhage, hemorrhagic diathesis, or patients under anticoagulation therapy, the best available evidence supports that deep vein thrombosis (DVT) and venous thromboembolism (VTE) prophylaxis can safely be initiated within 48–72 h of admission for blunt splenic trauma, regardless of injury grade, without concern for exacerbation of bleeding, or failure of NOM **[Quality of Evidence: Moderate]**. Occurrence rates of thrombocytosis in patients after SAE seem comparable to patients who undergo splenectomy, in contrast with average platelet values in those managed with observation alone. So, SAE may be an independent risk factor for thrombotic events in WSES Class II–III (AAST Grades III–V) blunt splenic injuries **[Quality of Evidence: Moderate]**. Regarding the choice between low molecular weight heparin (LMWH) versus unfractionated heparin (UH), indirect evidence coming from trauma cases in general, and liver trauma specifically, suggests that LMWH is superior to UH for DVT and VTE prevention and may additionally reduce pulmonary embolism (PE) and mortality **[Quality of Evidence: Low]**	**Recommendation:** For patients with blunt splenic injuries treated with NOM with/without splenic artery angioembolization (SAE), in the absence of specific complications, the panel suggests that DVT and VTE prophylaxis with LMWH be started within 24 h from hospital admission for patients with WSES Class I (AAST Grades I–II) and within 48–72 h for those with WSES Class II–III (AAST Grades III–V) splenic injuries **[Strength of Recommendation: Conditional recommendation, based on Moderate Quality of Evidence, Agreement 91.3%]**
**Research Question: 3.** How long should patients with splenic trauma treated with NOM be followed up in the hospital according to the injury grade?	
**Priority level:** *High*	
**Statement:** For patients treated with NOM for splenic injuries, most NOM failures occur early, primarily in the first 24 h. The risk of NOM failure and subsequent splenectomy is highest in the first 24–72 h of admission, with only 3% of splenectomies occurring later in the hospital course. Readmission is relatively rare in patients treated with NOM and most often occurs within 7 days of discharge **[Quality of Evidence: Moderate]**. Length of hospital stay for children with isolated splenic injuries could be based upon clinical presentation and hemodynamic status, as there is insufficient evidence to support the use of injury grade as the unique determinant of the stay **[Quality of Evidence: Moderate]**. Family and patient education post-discharge could be considered to reduce the readmission rate **[Quality of Evidence: Moderate]**	**Recommendation:** The panel suggests 1 day (for low-grade splenic injuries—WSES Class I, AAST Grades I–II) to 3 days (for high-grade splenic injuries—WSES Classes II–II, AAST Grades III–V) of hospital admission, with the duration of stay based on hemodynamic status, hemoglobin and hematocrit stability, and results of the follow-up CEUS/CT scan at 48–72 h for adult patients **[Strength of Recommendation: Conditional recommendation based on Moderate quality of evidence, Agreement 87%]**. Admission to a monitored setting (high dependency unit—HDU—or intensive care unit—ICU) is suggested for adult patient with high-grade splenic injuries treated with NOM **[Strength of Recommendation: Conditional recommendation based on Low quality of evidence, Agreement 87%]**. The panel suggests that early discharge after NOM for blunt splenic injury, especially those with WSES Classes II–III (AAST Grade ≥ III), could be at least accompanied by an explicit patient and caregiver education regarding the risk of outpatient rupture and, in every case, an outpatient clinical follow-up, telephone, GP-follow-up, or community nurse follow-up after 5–7 days is recommended **[Strength of Recommendation: Conditional recommendation based on Moderate quality of evidence, Agreement 93.5%]**. The panel suggests abandoning strategies according to which the length of hospital stay is injury AAST Grade plus 1 day in children with splenic injuries treated with NOM in favor of an approach based on hemodynamic status, hemoglobin and hematocrit stability **[Strength of Recommendation: Conditional recommendation based on Moderate quality of evidence, Agreement 89.1%]**
**Research Question: 4.** What kind of hemodynamic monitoring is indicated during NOM for splenic trauma according to the injury grade?	
**Priority level:** *Medium*	
**Statement:** No studies focused on hemodynamic monitoring in patients with splenic injuries treated with NOM. No comparative studies focused on the proper monitoring according to the grade of the injury. Consequently, the evidence available is derived from case series and studies designed for other purposes, making the quality of evidence very low. Hemodynamic monitoring in patients with high-grade injuries is performed with continuous monitoring of vital parameters (pulse pressure, cardiac frequency and peripheral O2 saturation), frequent medical and nursing monitoring, and frequent evaluation of serum hemoglobin and hematocrit **[Quality of Evidence: Very low]**	**Recommendation:** There is not enough evidence to recommend specific hemodynamic monitoring in patients with splenic injuries treated with NOM. The panel suggests that all patients treated with NOM for high-grade splenic injuries (WSES Classes II–III, AAST Grade ≥ III) might receive continuous hemodynamic monitoring of vital parameters (pulse pressure, cardiac frequency, and peripheral O2 saturation) and frequent serum hemoglobin and hematocrit levels evaluation (every 8 h) **[Strength of Recommendation: Conditional recommendation, based on Very low quality of evidence, Agreement 93.5%].** In patients with low-grade splenic injuries and stable hemodynamic status (WSES Class I, AAST Grades I–II) treated with NOM, the panel suggests close medical and nursing monitoring with evaluations of hemoglobin and hematocrit levels every 12–24 h if no complication occurs **[Strength of Recommendation: Conditional recommendation, based on Very low quality of evidence, Agreement 91.3%]**
**Research Question: 5.** When is splenic artery embolization (SAE) indicated for patients treated with NOM for splenic trauma?	
**Priority level:** *Urgent*	
**Statement:** In stable patients with high-grade splenic injuries (WSES Classes II–III, AAST Grade ≥ III), splenic artery angioembolization (SAE) represents an effective adjunctive tool to NOM, reducing the failure of the conservative approach and the need for surgery. Angiography and SAE should be performed at an early stage when contrast extravasation or vascular injuries (pseudoaneurysm, arteriovenous fistula formation, vessel truncation) are detected on admission CT scan, as well as in high-grade splenic injuries (WSES Class III, AAST Grades IV–V) even if contrast extravasation is not detected. However, small pseudoaneurysms can be safely observed without SAE [**Quality of Evidence: High**]. Some discrepancies in the management of WSES Class II (AAST Grade III) blunt splenic injury without contrast extravasation still exist among the experts. However, based on the available evidence, SAE in WSES Class II (AAST Grade III) splenic injury without vascular extravasation cannot be currently recommended as routine practice. In children, SAE has not been shown to be efficacious [**Quality of Evidence: Low**]. The available literature is inconclusive regarding whether proximal or distal embolization should be used to avoid significant re-bleeding, and larger prospective cohort studies are required. However, both techniques have an equivalent rate of post-procedural splenic infarctions and infections. Minor complications occur more often after distal embolization. This is primarily explained by the higher rate of segmental infarctions after distal embolization [**Quality of Evidence: Low**]	**Recommendation:** The panel suggests splenic artery angioembolization (SAE) as the first-line intervention in patients with hemodynamic stability and arterial blush on CT scan, irrespective of injury grade, where the expertise and resources required to carry out the procedure are readily available **[Strength of Recommendation: Conditional recommendation, based on Moderate quality of evidence, Agreement 93.5%]**. A low threshold for SAE is suggested for patients with WSES Class II (AAST Grade III) blunt splenic injury without contrast extravasation in the presence of risk factors for NOM failure (i.e., age above 55 years old, high injury severity score, the need for red cell transfusions in ED or during the first 24 h, patients on anticoagulant therapy, HIV disease, cirrhosis, and drug addiction) **[Strength of Recommendation: Conditional recommendation, based on Low quality of evidence, Agreement 89.1%]**. The panel suggests angiography and eventual SAE in all hemodynamically stable adult patients with WSES Class III (AAST Grades IV–V) splenic injuries, even in the absence of CT blush, in centers with adequate experience and where SAE is rapidly available, especially when concomitant surgery that requires change of position and that may cause dislodgement of clots and rebleeding (i.e., spinal surgery in the prone position) is needed **[Strength of Recommendation: Conditional recommendation, based on Moderate quality of evidence, Agreement 83.7%]**. The panel suggests SAE be reserved for children who demonstrate evidence of ongoing bleeding with a vascular blush seen on CT **[Strength of Recommendation: Conditional recommendation, based on Low quality of evidence]**. The panel suggests preferring proximal SAE over distal SAE when splenic artery angioembolization is needed **[Strength of Recommendation: Conditional recommendation, based on Low quality of evidence, Agreement 84.8%]**
**Research Question: 6.** Is there a need for radiological follow-up during the hospital stay for patients treated with NOM for splenic trauma according to the injury grade?	
**Priority level:** *Urgent*	
**Statement:** A selective re-imaging strategy appears safe, as re-imaging asymptomatic patients rarely results in intervention. Although limited due to the lack of high-quality research, a follow-up CT scan seems to be justified only in patients with WSES Class II (AAST Grade III) or higher splenic injuries to identify vascular abnormalities after splenic trauma. Indeed, 75–90% of adults with pseudoaneurysm or arteriovenous fistula and all such lesions in the pediatric population occur in patients with injuries of such grade [**Quality of Evidence: Low**]. In higher grade injuries (WSES Classes II–III, AAST Grades III–V), contrast-enhanced ultrasound (CEUS)/CT scan follow-up might be performed in the first 48–72 h to exclude the development of vascular complications [**Quality of Evidence: Low**]. In literature, most delayed splenic complications are diagnosed in the first 7 days from trauma and rarely in AAST Grade I splenic injuries [**Quality of Evidence: Low**]	**Recommendation:** The panel suggests radiological follow-up to be based on clinical findings in WSES Class I (AAST Grades I–II) splenic trauma treated with NOM and suggests against routine imaging follow-up in these patients **[Strength of Recommendations: Conditional recommendation based on Low quality of evidence, Agreement 91.3%]**. The panel suggests repeating imaging with contrast-enhanced ultrasound (CEUS)/CT scan in 48 h to 72 h post-admission and, eventually, at 5–7 days of trauma (only if remarkable changes in CT scan at 72 h are detected, or new signs/symptoms related to the trauma occur) in adult patients with WSES Class II splenic injuries (AAST Grade III) or higher treated with NOM, regardless of whether SAE has been performed or not **[Strength of Recommendation: Conditional recommendation, based on Low quality of evidence, Agreement 82.6%]**
**Research Question: 7.** What is the best imaging method to follow-up patients treated with NOM for high-grade splenic injures in the acute phase during NOM (Ultrasound without contrast, Contrast-enhanced ultrasound, CT-Angio)?	
**Priority level:** *High*	
**Statement:** Although contrast-enhanced CT scan is the gold standard modality for imaging abdominal organ traumatic injury, contrast-enhanced ultrasound (CEUS) has developed a role in the follow-up of traumatic injuries, including splenic trauma. CEUS is a valuable tool for detecting post-traumatic lesions; it is comparable to CT after splenic embolization and may replace CT in follow-up studies in expert hands [**Quality of Evidence: Low**]. CEUS may be considered in children, although repeat imaging in children is rarely needed [**Quality of Evidence: Low**]	**Recommendation:** The panel suggests, in expert hands and dedicated institutions, using CEUS as an alternative imaging modality in the follow-up of conservatively managed splenic trauma to reduce the number of CT examinations, especially in children [**Strength of Recommendation: Conditional recommendation, based on Low quality of evidence, Agreement 91.3%**]
**Research Question: 8.** Should patients treated with NOM for splenic trauma (with or without splenic artery embolization) receive vaccinations?	
**Priority level:** *Medium*	
**Statement:** Most patients with splenic injuries treated with NOM, including those treated with splenic artery embolization (SAE), present a significantly lower rate of post-traumatic infections than those who undergo splenectomy **[Quality of Evidence: Moderate]**. SAE does not show higher rates of early and delayed infective complications compared with NOM without SAE **[Quality of Evidence: Low]**. There is insufficient evidence to advise mandatory vaccination in patients treated with NOM for splenic trauma, either with or without SAE **[Quality of Evidence: Moderate]**	**Recommendation:** The panel suggests against routine vaccination for overwhelming post-splenectomy infection (OPSI) from encapsulated bacteria in patients treated with NOM for splenic injury with or without SAE [**Strength of Recommendation: Conditional recommendation, based on Moderate quality of evidence, Agreement 89.1%**]. The panel suggests a tailored approach driven by the immunologic state of the patient before the splenic injury and taking into account possible effects of SAE in losing 50% or more of spleen mass. If 50% or more of the splenic mass is lost, and in every case of WSES Class III (AAST Grade V) injury, patients might be considered as asplenic and potentially more susceptible to OPSI; therefore, they could receive immunization against encapsulated organisms [**Strength of Recommendation: Conditional recommendation, based on Low quality of evidence, Agreement 93.5%**]
**Research Question: 9.** For how long should patients with splenic trauma treated with NOM be followed up after hospital discharge according to the injury grade?	
**Priority level:** *Medium*	
**Statement:** Risk factors for late failure of NOM and hospital readmission include hemoperitoneum with signs of blush at first contrast-enhanced CT and high (WSES Class III, AAST IV–V) grade injuries **[Quality of Evidence: Low]**. There is neither agreement nor sufficient evidence to recommend the optimal length of follow-up after hospital discharge in patients with splenic trauma treated with NOM. The role of imaging has not been cleared out in the post-discharge follow-up **[Quality of Evidence: Very low]**. Imaging follow-up, either by CEUS or CT scan, does not seem to provide clinical benefits in the post-discharge period, and it might be considered in severe injuries (WSES Class III, AAST Grades IV–V) and depending on the level of activity (professional athletes, those practicing high-impact sports, heavy lifting) **[Quality of Evidence: Very low]**. Before returning to major physical activity, imaging follow-up with a contrast-enhanced CT scan or CEUS seems to be indicated to assess the status of the healing process after WSES Class II–III (AAST Grades III–V) splenic injuries treated with NOM **[Quality of Evidence: Low]**. The panel suggests that patients with blunt splenic injuries treated with NOM (with or without SAE) and families be informed of long-term complications and the possibility of NOM failure **[Quality of Evidence: Low]**	**Recommendation:** The panel suggests selective imaging follow-up at one, three, and (unless imaging confirms healing at 3 months) 6 months after discharge for patients with blunt splenic injuries treated with NOM only in the presence of risk factors for long-term complications and depending on the level of activity (professional athletes, those practicing high-impact sports, heavy lifting). The choice to perform imaging follow-up after discharge includes several considerations, such as the presence of severe splenic injuries (WSES Class III, AAST Grades IV–V); the association with other injuries that would warrant other specific follow-up; the age and expected activity level of the patient post-discharge; the type of NOM utilized (e.g., strictly observational or including interventional radiology); the duration of the hospital stay (with earlier discharge at risk of higher readmission rates) **[Strength of Recommendation: Conditional recommendation, based on Low quality of evidence, Agreement 86.0%]**. The panel suggests performing contrast-enhanced imaging follow-up (CT/CEUS) before returning to major physical activity (2–4 months in high-grade injuries) in adult patients with WSES Class II–III (AAST Grades III–V) splenic injuries treated with NOM **[Strength of Recommendation: Conditional recommendation, based on Low quality of evidence, Agreement 89.1%]**. Pediatric patients could be offered follow-up as outpatient consultation focusing on psychological response to injury and pain management, with further radiological examinations only if clinically indicated **[Strength of Recommendation: Conditional recommendation, based on Very low quality of evidence, Agreement 95.7%]**. The panel suggests imaging follow-up with CEUS before return to major physical activity in children with WSES Class II–III (AAST Grades III–V) splenic injuries treated with NOM **[Strength of Recommendation: Conditional recommendation, based on Low quality of evidence, Agreement 82.6%]**
**Research Question: 10.** How long should patients treated with NOM for splenic trauma abstain from major physical activities based on the grade of the splenic injury?	
**Priority level:** *High*	
**Statement:** Although there is a lack of high-quality research on the duration and intensity of restricted activity and return to play after blunt spleen injuries treated with NOM, the current trend is toward shorter post-discharge follow-up with earlier return to daily activity. Specifically, physical activity restrictions limited to 4 weeks after the injury, irrespective of injury grade on CT, seem safe in pediatric patients. Non-contact activity, including school, can be allowed safely after discharge [**Quality of Evidence: Low**]	**Recommendation:** The panel suggests that major activity restrictions (athletic activities, no-contact sports, heavy lifting) in adults with splenic trauma treated with NOM might be prescribed for 3–5 weeks in low-grade splenic injuries (WSES Class I, AAST Grades I–II) and up to 2–4 months in high-grade injuries (WSES Classes II–III, AAST Grades III–V). A follow-up imaging with contrast-enhanced CT/CEUS is suggested before return to full activities to confirm healing [**Strength of Recommendation: Conditional recommendation based on Low quality of evidence, Agreement 89.1%**]. The panel suggests that major activity restrictions in children with splenic trauma treated with NOM could be limited to 4 weeks after the injury, irrespective of injury grade on CT scan [**Strength of Recommendation: Conditional recommendation based on Low quality of evidence, Agreement 87%**]
**Research Question: 11.** Which is the best treatment of post-splenic artery embolization (SAE) necrosis of the spleen for patients with splenic trauma treated with NOM? Surgery or radiological drainage?	
**Priority level:** *High*	
**Statement:** Splenic abscess following NOM for splenic injuries is an infrequent complication. In the case of a splenic abscess, surgery and percutaneous drainage seem similar in safety and efficacy, but the quality of supporting evidence is poor **[Quality of Evidence: Low]**	**Recommendation:** The panel suggests considering percutaneous drainage as the first-line treatment of splenic abscesses after NOM in case of availability of the interventional radiology technique, adequate skills, and technical feasibility **[Strength of Recommendation: Conditional recommendation based on Low quality of evidence, Agreement 95.7%]**. In case of failure of percutaneous drainage (intense and persistent pain in patients with sepsis) or unavailability, the panel suggests performing splenectomy, open or laparoscopic, according to local expertise **[Strength of Recommendation: Conditional recommendation based on very Low quality of evidence, Agreement 97.8%]**

*WSES* World Society of Emergency Surgery, *AAST* American Association for the Surgery of Trauma

### Notes on the use of this consensus paper

This consensus paper presents the diagnostic and therapeutic methods for optimal follow-up strategies for patients with blunt splenic trauma treated with NOM. The practice suggestions promulgated in this manuscript do not represent a standard of practice. These are suggested plans of care based on the best available scientific evidence and experts' consensus, but they do not exclude other approaches as being within the standard of practice. They should not be used to compel adherence to a given medical management method, which method should be finally determined after considering the conditions at the relevant medical institution (staff levels, experience, surgical skills, equipment, etc.) and the characteristics of the individual patient. The treatment results' responsibility rests with those directly engaged and not with the consensus group.

**Research Question 1:** What is the optimal duration of bed rest for patients treated with NOM for splenic trauma according to the injury grade? (***High priority***).

**Statement.** Abbreviated bed rest of 24 h is safe and does not increase NOM failure. The panel agrees that early mobilization could be considered in all splenic injury patients according to their general condition and associated injuries **[Quality of Evidence: Moderate]**. The day on which mobilization was performed is not associated with an increased risk of delayed hemorrhage in adult patients with low (WSES Class I, AAST Grades I–II) and high-grade (WSES Classes II–III, AAST Grades III–V) splenic injuries **[Quality of Evidence: Moderate]**. Current evidence supports an abbreviated period of bed rest of 24 h or less after splenic injury for hemodynamically stable children whose hemoglobin has been documented to be stable **[Quality of Evidence: Moderate]**.

**Recommendation.** The panel suggests allowing early mobilization within 24 h in patients with low-grade (WSES Class I, AAST Grades I–II) splenic injuries treated with NOM. Patients with WSES Class II (AAST Grade III) splenic injuries can be mobilized after 2 days from the trauma if no other contraindications exist **[Strength of Recommendation: Conditional recommendation, based on Low quality of evidence, Agreement 89.1%]**. In patients with high-grade splenic injuries (WSES Class III, AAST Grades IV–V), if no other contraindications to early mobilization exist, the patient can be mobilized safely after 2 days from the trauma when three successive hemoglobins 8 h apart after the first are within 10% of each other, and if clinical parameters remain stable **[Strength of Recommendation: Conditional recommendation, based on Low quality of evidence, Agreement 87.2%]**. The panel suggests a shortened protocol of one night of bed rest for WSES Class I injuries (AAST Grade II; no need for bed rest for AAST Grade I) and two nights for WSES Classes II–III (AAST Grade ≥ III) in children when clinical parameters remain stable **[Strength of Recommendation: Conditional recommendation, based on Moderate quality of evidence, Agreement 82.6%]**.

In patients with blunt solid organ injuries, the panel suggests to weigh the risk of delayed hemorrhage against the risk of prolonged bed rest, which includes deep vein thrombosis (DVT), venous thromboembolism (VTE), pulmonary embolism (PE), pneumonia, hospital infections, and is associated with increased length of hospitalization and costs. Historically, NOM for solid organ injuries included bed rest to prevent delayed hemorrhage because movement or an unintended patient fall could disrupt the clot overlying a splenic or liver injury and lead to a delayed organ rupture, even up to many days after the initial trauma. However, during the last 10 years, this supposition has been contested in pediatric trauma patients [17], and protocols incorporating periods of strict bed rest have proven unnecessary. Several studies demonstrated that a shortened bed rest of 24 h is safe, does not increase failure of NOM, and that the day on which mobilization was commenced is not associated with an increased risk of delayed hemorrhage both in low (Grades I–II) and high (Grades III–V) grade splenic injuries [17, 18]. Failure of NOM is principally due to the evolution of PSA or artero-venous fistula, and early mobilization does not affect bleeding from these vascular injuries.

Our systematic review of the literature regarding this topic retrieved seven studies, of which four were conducted in the pediatric population [19–22], and three in the adult population [17, 23, 24]. Two were prospective cohort studies [19, 20], five were retrospectives [5, 17, 21, 23, 24], and one was a systematic review of the literature [22].

Based on the current evidence, the timing of mobilization of patients with blunt solid organ injuries does not seem to contribute to delayed hemorrhage requiring laparotomy. Therefore, protocols incorporating prolonged periods of strict bed rest are unnecessary. In the retrospective study by London et al. [24], of 454 patients with blunt solid organ injuries admitted to the hospital for NOM, 4.0%, 1.0%, and 7.1% for renal, hepatic, and splenic injuries, respectively, failed conservative treatment. Ten patients (5.5%) with splenic injuries failed secondary to delayed hemorrhage. Eighty-four percent of patients with renal injuries, 80% with hepatic injuries, and 77% with splenic injuries were mobilized within 72 h of admission. Day of mobilization was not associated with delayed splenic rupture in multivariate analysis (OR, 0.97; 95% CI 0.90–1.05). According to the authors, the timing of mobilization of patients with blunt solid organ injuries does not seem to contribute to delayed hemorrhage requiring laparotomy, as demonstrated by the fact that day of mobilization was not associated with delayed splenic rupture in multivariate analysis. Griffard et al. [23] evaluated the safety, in terms of risk of delayed hemorrhage and subsequent failure of NOM, and complications of early ambulation within 24 h in low-grade splenic trauma to assess the length of stay, development of pneumonia, pleural effusions, VTE, and PE. In this study, of the 163 total patients, 100 (61.3%) were on bed rest for at least 24 h after their injury, and 63 (38.7%) were ambulated within the first 24 h. In the group of patients allowed to ambulate during the first 24 h, there were no failures of NOM, whereas, in those for those who bed rest for at least 1 day was prescribed, 6 (6%) failed NOM. There were no statistically significant differences in DVT, PE, pleural effusions, pneumonia, and length of hospitalization.

Other studies evaluated the timing of mobilization of patients with blunt solid organ injuries concerning the rates of delayed rupture. They found that the timing of mobilization in these patients was not associated with an increased incidence of delayed rupture and hemorrhage. Teichman et al. [17] showed that a specific NOM protocol, including early mobilization in blunt splenic and hepatic injuries, is safe and more cost-effective than extended bed rest protocols. In their study, patients managed non-operatively from January 2008 through July 2011 were observed in the hospital under a prolonged bed rest protocol of 3 days prior to ambulation. In August 2011, an early mobilization protocol was adopted. Patients with low-grade splenic and/or hepatic injuries were observed with bed rest overnight until two successive hemoglobins 12 h apart were within 10% of each other. The patient was then ambulated and eligible for discharge if hemoglobin the morning following ambulation was within 10% of the previous values. Patients with high-grade injuries were admitted to the ICU and kept on bed rest until three successive hemoglobins 8 h apart after the first hemoglobin check were within 10% of each other. These patients were then ambulated and eligible for discharge if hemoglobin the following day was within 10% of the previous values. The authors found that the length of hospital stay was significantly reduced in the early mobilization group, with an average stay of 4.53 days in the prolonged bed rest group and 3.46 days in the early mobilization group. The two groups did not differ in NOM failure, angiography, embolization, or mortality. Through the implementation of an early mobilization protocol, the length of hospital stay was shortened by 1.07 days, and the cost of hospitalization was reduced by $7077, without any difference in NOM failure, angiography/embolization, or mortality.

In the pediatric population, the Trauma Committee of the American Pediatric Surgical Association (APSA) recommends a period of bed rest to equal the grade of injury plus 1 day [25]. However, the description of a shortened bed rest protocol for children was reported in 2008 by St Peter et al. [26]. They stated that a trauma protocol with overnight bed rest for AAST Grades I and II injuries and two nights for higher grades could be safely used. This shortened protocol was then validated in 2011 [19] in a prospective study that enrolled a total of 131 patients with blunt splenic or liver injuries and confirmed that a protocol of one night of bed rest for AAST Grade I and II injuries and two nights for Grade III or higher could be safely used, resulting in dramatic decreases in hospitalization compared with the APSA recommendations. Another study by St Peter et al. [20] further validated that an abbreviated protocol of one night of bed rest for AAST Grade I and II injuries and two nights for Grade ≥ III can be safely employed in children. In this validation study, injuries included isolated spleen in 130 (52%), liver only in 107 (43%), and both in 12 (5%). If the patient received a transfusion, the time frame was reset, and the clock for bed rest restarted. This study demonstrated that bed rest was the limiting factor keeping nearly two-thirds of their patients with splenic injuries in the hospital. If the authors had followed the APSA guidelines, these patients would have averaged an extra day of hospitalization. Dodgion et al. [21] confirmed that a shortened bed rest protocol consisting of 1-day bed rest for AAST Grade I–II splenic injuries and 2-days bed rest for Grade III or higher could safely reduce the duration of hospital stay without any increase in NOM failure and short-term complication rates. In a large cohort of 22,153 patients with blunt splenic or liver injuries, they found that application of an abbreviated bed rest protocol could potentially save 1.7 hospital days/patient or 36,964 patient hospital days nationally compared to the standard APSA protocol. According to the WSES guidelines [1], in hemodynamically stable children without a drop in hemoglobin levels, bed rest could be suggested for 24 h. Currently, there is no evidence to support bed rest as a treatment for blunt liver or splenic injuries, and no evidence suggests that bed rest prevents re-bleeding in children. Several prospective studies showed that a shortened period of bed rest is safe, even though prolonged bed rest protocols and admissions are still commonplace [22, 24].

**Research Question 2:** What is the optimal timing and type of anti-thrombotic prophylaxis for patients with splenic trauma treated with NOM? (***High priority***).

**Statements.** In the absence of contraindications related to associated injuries or conditions, such as intracranial hemorrhage, hemorrhagic diathesis, or patients under anticoagulation therapy, the best available evidence supports that deep vein thrombosis (DVT) and venous thromboembolism (VTE) prophylaxis can safely be initiated within 48–72 h of admission for blunt splenic trauma, regardless of injury grade, without concern for exacerbation of bleeding, or failure of NOM **[Quality of Evidence: Moderate]**. Occurrence rates of thrombocytosis in patients after SAE seem comparable to patients who undergo splenectomy, in contrast with average platelet values in those managed with observation alone. So, SAE may be an independent risk factor for thrombotic events in WSES Class II–III (AAST Grades III–V) blunt splenic injuries **[Quality of Evidence: Moderate]**. Regarding the choice between low molecular weight heparin (LMWH) versus unfractionated heparin (UH), indirect evidence coming from trauma cases in general, and liver trauma specifically, suggests that LMWH is superior to UH for DVT and VTE prevention and may additionally reduce pulmonary embolism (PE) and mortality **[Quality of Evidence: Low]**.

**Recommendation.** For patients with blunt splenic injuries treated with NOM with/without splenic artery angioembolization (SAE), in the absence of specific complications, the panel suggests that DVT and VTE prophylaxis with LMWH be started within 24 h from hospital admission for patients with WSES Class I (AAST Grades I–II) and within 48–72 h for those with WSES Class II–III (AAST Grades III–V) splenic injuries **[Strength of Recommendation: Conditional recommendation, based on Moderate Quality of Evidence, Agreement 91.3%]**.

Trauma patients are at high risk of deep venous thrombosis (DVT) and venous thromboembolism (VTE), and, without anti-thrombotic prophylaxis, more than 50% of patients may experience thrombotic complications [27, 28]. Thromboelastographic studies have identified a transition from trauma-induced coagulopathy to a hypercoagulable state at 48 h among patients with blunt solid organ injury, suggesting that 48 h might provide the optimal balance of minimizing the risk of both bleeding and VTE [27, 29]. Although DVT and VTE are not life-threatening complications, their association with pulmonary embolism (PE) carries potential morbidity and mortality. After major trauma, the reported mortality rate as a result of PE ranges from 0.4 to 50% [30, 31].

The Western Trauma Association (WTA) suggests starting VTE prophylaxis 24 h post-injury [32], whereas the Eastern Association for the Surgery of Trauma (EAST) recommends starting VTE prophylaxis within 48 h of injury [33]. Studies demonstrating the need for timely DVT prophylaxis initiation showed that a delay in initiation > 4 days is associated with a threefold VTE risk increase [34]. Several prospective and retrospective studies have demonstrated that VTE prophylaxis administration 24–48 h after arrival to the hospital is associated with reduced rates of VTE without increased need for blood transfusion or failure of NOM [35–39]. The 2017 WSES guidelines stated that LMWH-based prophylactic anticoagulation should be started as soon as possible from splenic trauma, including patients treated with NOM [1]. Our systematic review of the literature regarding this topic retrieved 15 studies, of which two were systematic reviews and meta-analyses [40, 41], two were prospective cohort studies [35, 38], and 11 were retrospective cohort studies [27, 28, 36, 37, 42–48]. To date, two systematic reviews and meta-analyses demonstrated that patients undergoing NOM for blunt solid organ injury could be safely and effectively prescribed early VTE prophylaxis. Murphy et al. [41] included ten studies comprising 14,675 patients that compared early (≤ 48 h) versus late (> 48 h) initiation of VTE chemoprophylaxis in adults with blunt splenic, liver, and/or kidney injury. All studies were non-randomized, and only one was prospective. The overall odds of failure of NOM were not different between early and late groups (OR 1.09; 95% CI 0.92–1.29), and there was no difference in the need for blood transfusion either during overall hospital stay (OR 0.91; 95% CI 0.70–1.18), or post-prophylaxis initiation (OR 1.23; 95%CI 0.55–2.73). The subgroup analysis focused on splenic injuries showed no difference in NOM failure (OR 0.91, 95% CI 0.42–1.97). Conversely, there were significantly lower odds of VTE when patients received early VTE prophylaxis (OR 0.51; 95% CI 0.33–0.81). In keeping with the results presented by Murphy et al., the most updated meta-analysis [40] found a non-statistically significant trend toward an increased risk of failure of NOM among patients receiving early VTE prophylaxis (OR 1.76, 95% CI 1.01–3.05), with no significant difference in risk of transfusion. Conversely, odds of DVT were significantly lower in the early group (OR 0.36, 95% CI 0.22–0.59) but without a difference in mortality (OR 1.50, 95% CI 0.82–2.75). However, in both the cited meta-analyses, all studies were at serious risk of bias due to confounding as they included only observational studies. For example, patients in the delayed VTE prophylaxis arm of each primary study had higher injury severity score (ISS) and AAST injury grades and more extended hospital and ICU admissions. Moreover, some studies included patients with head injuries, which are a conditional factor in deciding the timing of VTE prophylaxis on admission.

When looking at the primary studies included in the mentioned meta-analyses, patients were usually dichotomized into study groups based on VTE prophylaxis initiation time: early (≤ 48 h or ≤ 72) versus late (> 48 h or > 72 h). In the study by Rostas et al. [27], there were no enoxaparin-related hemorrhagic complications or hemorrhages necessitating operative intervention in any group. In Alejandro et al. [48], early use of LMWH was not associated with an increased rate of blood transfusions or an increased rate of failure of NOM. In the early group, two (4%) patients failed NOM compared with four (6%) patients in the late group (*P* = 0.593). The number of patients requiring blood transfusions within the first 5 days after admission was 25 (50.0%) in the early group and 36 (56.2%) in the late group (*P* = 0.507). The average number of blood units per patient within the first 5 days after admission was 3.2 ± 1.5 in the early group and 3.0 ± 1.8 in the late group (*P* = 0.782). In a retrospective study including patients aged 15 years or older who sustained blunt splenic, liver, and/or kidney injuries from January 2005 to December 2008, NOM failure rates and blood transfusion requirements were compared between patients who got LMWH early (≤ 3 days), patients who got LMWH late (> 3 days), and patients who did not receive LMWH [37]. After adjusting for demographic differences, the overall blood transfusion requirements for the early LMWH group were significantly lower than for patients with late LMWH administration (3.0 ± 5.3 units vs. 6.4 ± 9.9 units). When analyzing specifically the outcomes of patients with splenic injuries, the overall failure rate of NOM was 7.8% and was not different between the early, late, and no LMWH group after adjustment for differences in demographics. Of those receiving LMWH early, 50.0% had a high-grade lesion, and these patients showed a similar failure rate compared with high-grade injured patients who received LMWH late or those who did not receive LMWH. Similarly, in another retrospective cohort study, prophylactic LMWH or UH was administered at two different time intervals (within 72 h after admission or 72 h after admission) in patients with blunt solid organ injuries. In contrast, one group was not put under heparin at all. Although not statistically significant, VTE was more frequent in the no heparin group compared to the early (≤ 72 h) and late (> 72 h) heparin groups (10.8 vs. 4.8 vs. 1.3%). Failed NOM occurred only in patients with splenic injuries and was significantly more frequent in the no heparin group than in the early and late heparin group. Also, Joseph et al. [45] reported that early enoxaparin-based anticoagulation might be a safe option in trauma patients with blunt solid organ injury. They found no difference in hemorrhagic complication and NOM failure rate in patients with early (< 48 h), intermediate (48–72 h), and late (> 72 h) VTE prophylaxis. Conversely, there was a trend toward a higher incidence of thromboembolic complications in patients with late initiation of prophylaxis. In Schellenberg et al. [35], early prophylaxis patients had significantly fewer DVT but similar rates of PE compared to late prophylaxis patients. Traumatic brain injury was the only significant risk factor for late prophylaxis, and no patient in either group required delayed intervention (operative or interventional radiology) for bleeding. There was also no difference in the volume of post-prophylaxis blood transfusion. Similarly, Griffard et al. [38] demonstrated that administering DVT prophylaxis sooner than 48 h does not increase failure of NOM of AAST Grade III–V splenic injuries. In their prospective study, the authors included 104 high-grade (AAST III–V) splenic trauma patients who received prophylaxis with UH or LMWH within 72 h of diagnosis. They observed six failures of NOM, and there was no statistically significant difference between the < 24 h and > 24 groups or between the < 48 h and 48–72 h groups. Moreover, in the linear regression analysis model describing the time to initiation of DVT prophylaxis using age, sex, splenic injury grade, and ISS, the NOM failure rate decreased by 0.00002% for each hour prior to giving DVT prophylaxis (*P* = 0.111). From this, the authors proposed a non- inferiority statement that initiating DVT prophylaxis within 48 h does not increase the failure rate compared to patients receiving DVT prophylaxis between 48 and 72 h. The results reported by Kwok et al. [43] corroborated the statement that early initiation within < 48 h of VTE prophylaxis is safe in patients with blunt splenic injuries treated non-operatively, but also it may be safe as early as 24 h. Lin et al. [46] explored the American College of Surgeons TQIP database from 2013 to 2014 to identify adult patients who underwent NOM for isolated AAST Grade III–V blunt splenic injuries. The incidence of NOM failure after the initiation of VTE prophylaxis was compared between two groups: VTE prophylaxis ≤ 48 h after admission (early prophylaxis group) and > 48 h (late prophylaxis group). There was no significant difference in the NOM failure rate after initiating VTE prophylaxis between the early and late prophylaxis groups. In the multiple logistic regression analysis, early initiation of VTE prophylaxis was not significantly associated with NOM failure. Results of the subgroup analysis in patients with Grade IV–V splenic injuries also suggest that early administration of VTE prophylaxis is not significantly associated with an increased incidence of NOM failure. Skarupa et al. [36] performed a 2-year retrospective analysis of 36,187 patients from the American College of Surgeons TQIP, including all adult trauma patients with blunt solid organ injuries who underwent NOM. Patients were stratified into three groups based on the timing of VTE chemoprophylaxis (early, ≤ 48 h of injury; late, > 48 h of injury; and no prophylaxis group). After controlling for confounders, patients receiving early prophylaxis had lower rates of DVT and PE than the no prophylaxis and late prophylaxis groups, but no difference between the three groups regarding the post-prophylaxis blood transfusions, failure of NOM, and mortality was reported. Also, in the study by Khatsilouskaya et al. [44], early heparin administration within 72 h from hospital admission was not associated with an increased NOM failure rate or higher in-hospital mortality. According to Gaitanidis et al. [47], thromboprophylaxis within 48 h should be considered in patients with blunt solid organ injuries undergoing NOM who are at low likelihood of bleeding. Conversely, in the presence of a higher likelihood of bleeding, along with a history of diabetes mellitus, or in the case of splenic and high-grade liver injuries, the authors suggest an intermediate delay (48–72 h) of thromboprophylaxis.

Wernick et al. [49] evaluated hematologic parameters after splenic trauma in patients who underwent splenectomy, SAE, or observation. They found occurrence rates of thrombocytosis in patients post-SAE comparable to patients who underwent splenectomy, in contrast with normal platelet values in those managed with observation alone. SAE, as reported by Lewis et al. [42], may be an independent risk factor for thrombotic events in AAST Grade III–V blunt splenic injuries. In keeping with these findings, in a retrospective review of the American College of Surgeons TQIP of 2643 high-grade (AAST III–V) splenic trauma patients managed with NOM, the incidence of DVT was 4.5% in patients who underwent SAE, compared to 1.4% in patients who did not. In the multivariable analysis, angioembolization was an independent risk factor for DVT (OR 2.65) and any VTE (OR 2.04). Moreover, analysis of splenic injury grade showed that angioembolization remained an independent risk factor for DVT in Grades IV–V and VTE in Grade III injuries. In the same study, initiation of pharmacological VTE prophylaxis 48 h after admission was associated with increased VTE rates compared to early initiation (OR 1.75) [31, 42].

Regarding the choice between LMWH versus UH, indirect evidence from general trauma cases and liver trauma suggests that LMWH is superior to UH for DVT and VTE prevention and may additionally reduce PE and mortality. A recent systematic review and meta-analysis [50] that included four randomized trials for a total of 879 patients and eight observational studies (306,747 patients) found that, based on pooled randomized data, LMWH reduces DVT (RR 0.67, 95% CI 0.50–0.88, moderate certainty) and VTE (RR 0.68, 95% CI 0.51–0.90, moderate certainty) compared to UH. Moreover, compared to UH, LMWH may reduce PE (adjusted OR 0.56, 95% CI 0.50–0.62) and mortality (adjusted OR 0.54, 95% CI 0.45–0.65). In keeping with the results reported by Tran et al. [50], Jakob et al. [51] analyzed data of 4074 patients with blunt isolated severe liver injuries selected for NOM who received VTE prophylaxis with either UH or LMWH. Notably, LMWH was independently associated with lower mortality (OR 0.36) compared to UH. A 2013 Cochrane review that included 16 randomized controlled trials with more than 3005 patients found LMWH appeared to reduce the risk of DVT compared to UH (RR 0.68; 95% CI 0.50–0.94) [52].

**Research Question 3:** How long should patients with splenic trauma treated with NOM be followed up in the hospital according to the injury grade? (***High priority***).

**Statement:** For patients treated with NOM for splenic injuries, most NOM failures occur early, primarily in the first 24 h. The risk of NOM failure and subsequent splenectomy is highest in the first 24–72 h of admission, with only 3% of splenectomies occurring later in the hospital course. Readmission is relatively rare in patients treated with NOM and most often occur within 7 days of discharge **[Quality of Evidence: Moderate]**. Length of hospital stay for children with isolated splenic injuries could be based upon clinical presentation and hemodynamic status, as there is insufficient evidence to support the use of injury grade as the unique determinant of the stay **[Quality of Evidence: Moderate]**. Family and patient education post-discharge could be considered to reduce the readmission rate **[Quality of Evidence: Moderate]**.

**Recommendation.** The panel suggests 1 day (for low-grade splenic injuries—WSES Class I, AAST Grades I–II) to 3 days (for high-grade splenic injuries—WSES Classes II–II, AAST Grades III–V) of hospital admission, with the duration of stay based on hemodynamic status, hemoglobin and hematocrit stability, and results of the follow-up CEUS/CT scan at 48–72 h for adult patients **[Strength of Recommendation: Conditional recommendation based on Moderate quality of evidence, Agreement 87%]**. Admission to a monitored setting (high dependency unit-HDU- or intensive care unit-ICU-) is suggested for adult patient with high-grade splenic injuries treated with NOM **[Strength of Recommendation: Conditional recommendation based on Low quality of evidence, Agreement 87%]**. The panel suggests that early discharge after NOM for blunt splenic injury, especially those with WSES Classes II–III (AAST Grade ≥ III), could be at least accompanied by an explicit patient and caregiver education regarding the risk of outpatient rupture and, in every case, an outpatient clinical follow-up, telephone, GP-follow-up, or community nurse follow-up after 5–7 days is recommended **[Strength of Recommendation: Conditional recommendation based on Moderate quality of evidence, Agreement 93.5%]**. The panel suggests abandoning strategies according to which the length of hospital stay is injury AAST Grade plus 1 day in children with splenic injuries treated with NOM in favor of an approach based on hemodynamic status, hemoglobin and hematocrit stability **[Strength of Recommendation: Conditional recommendation based on Moderate quality of evidence, Agreement 89.1%]**.

The failure rate of NOM has been described in multiple reports after 2000 to range between 4 and 12%, with lower incidence in Level I trauma centers [4, 53]. Several risk factors of NOM failure have been reported, including the need for red cell transfusions in the emergency department or during the first 24 h [11, 54], hemoglobin and hematocrit levels at admission [11], HIV disease, cirrhosis, and drug addiction [55]. The presence of a blush at CT scan has been considered a risk factor for NOM failure only in studies in which SAE was not adopted [56], whereas the extension of hemoperitoneum at imaging alone cannot be considered an absolute contraindication for NOM [4, 57–59]. Higher injury grades and ISS are strictly associated with the risk of NOM failure [11, 60]. Moreover, it has been suggested that age > 55 years could be a risk factor for NOM failure only in high injury grades [61, 62]. Most patients who fail NOM do so within the first 3 days following trauma. However, the time to failure varies considerably. According to Clancy et al. [63], the median time to surgery for patients who failed NOM was 11.2 h.

In a 2008 study by Smith et al. [53], of patients who failed NOM, a full 95% failed within 72 h, and failure was highly related to the grade, with decreasing success of NOM noted at AAST Grade III and above. Additional observation for 48 h yielded only another 1.5% failure rate. Another 2008 retrospective cohort study of 449 patients by McCray et al. [5] demonstrated similar findings. Failure was again highly related to the grade of injury, and all patients with AAST Grade I injury had successful NOM. This continued as 99% for Grade II and 94% and 84% for grade III and IV, respectively. As in prior studies, the vast majority of NOM failures occurred early on, primarily in the first 24 h. All Grade I patients stayed in the hospital for at least 24 h, and all other injury grades had a 36-plus hour admission. NOM failure occurred within 3 days of injury for the majority of patients also in extensive database studies [11, 12]. Peitzman et al. [11] reported that most NOM failures occurred within the first 24 h (60%) with decreasing frequency on subsequent days (14% on the second day, 7% on the third day), so that 80% of failures occurred within the first 72 h. Zarzaour et al. [64], in a Tennessee statewide database analysis from 2000 to 2005, found that, of 1932 patients discharged home after NOM of splenic injuries, 27 (1.4%) were re-admitted for splenectomy within 180 days. The median time from injury to re-admission for splenectomy was 8 days, with an overall 180-day risk of splenectomy of 1.4% after NOM and discharge home. Similarly, in the study by Santorelli et al. [65], of 15,596 patients from the US National Readmission Database from 2016 to 2017, the spleen-related readmission rate after NOM was 2.4%, with the majority (70%) occurring within 7 days of discharge.

In the 2017 retrospective, cross-sectional study by Smith et al. [66], of 154 adult patients with blunt splenic injury treated with NOM, 16.1% of patients re-bled following NOM without SAE in a median of 2.3 days, whereas 28.6% of patients re-bled in a median of 2.0 days. Grade III–V injuries were a significant predictor of the failure of NOM ± SAE (OR 15.6, 95% CI 3.1–78.9, *P* = 0.001). No Grade I injuries and only 3.3% Grade II injuries re-bled following NOM. So, the authors concluded that AAST Grade I and II patients could be discharged after 24 h with appropriate advice. Zarzaur et al. [67] showed that splenectomy for NOM failure after 24 h is rare. Only 12 patients of 383 enrolled in the study (3.1%) underwent splenectomy between 24 h and 9 days after injury, whereas 0.27% required readmission for delayed rupture on post-injury day 12. Notably, no Grade I injuries experienced this type of complication. So, the splenectomy rate after 24 h of NOM was 1.5 per 1000 patient-days, and after the initial 24 h, no additional interventions were warranted for patients with Grade I injuries. In Liechti et al. [68], failure of NOM occurred in 3.9% of patients, and every failure of NOM was detected following the clinical deterioration in the first 48 h.

The only randomized controlled trial focusing on the safety and feasibility of early discharge in adult patients with blunt liver or splenic trauma was published recently by Kumar et al. [69]. This pilot trial focused on AAST Grades I–III trauma patients. Patients aged 18–60 years with blunt splenic injuries planned for NOM were randomized into the experimental group for which discharge was planned on day three if pre-planned discharge criteria were met (hemodynamic stability, no or minimum pain, no requirement for additional blood transfusion, no gross increase in hemoperitoneum), and the control group (discharge day 5). Both groups included 30 patients who were comparable in demography and injury-related parameters. Twenty-seven patients (90%) from the experimental group and 28 from the control group (93%) were discharged on the proposed day. Three patients in the control group had unplanned hospital visits for reasons unrelated to the traumatic injury.

Nowadays, pediatric solid organ injury management could be guided by hemodynamic status and not only by injury grade on CT scan. The 2000 American Pediatric Surgical Association (APSA) trauma committee [25] directed the treatment for each grade of injury and suggested the length of hospital stay be grade plus 1 day. Mehall et al. [70] reported the first prospective study directly challenging the APSA guidelines shortly after they were introduced. The discharge occurred 48 h post-injury if patients had no abdominal tenderness, tolerated a regular diet, and had a stable hematocrit. All patients were managed non-operatively without transfusion, and 43 of 44 completed the protocol, with an in-hospital mean observation time of 55.2 ± 12.3 h. The systematic review by Gates et al. [71] included 31 articles (four prospective studies and 27 retrospective studies). The total number of blunt splenic injuries reported was 18,105, with predominantly AAST Grade II, III, and IV injuries. The median length of hospitalization was 3.9 (range 1.2–10.8) days, and the median length of ICU stay was 2.0 days (range 0–6.3). In this review, studies that correlated solid organ injuries and length of hospital stay (LOS) concluded that LOS should be based upon hemodynamic stability rather than grade alone. McVay et al. [72] in 2008 proposed a management protocol for pediatric patients with blunt liver and splenic injuries based on hemodynamic status. Using this protocol, the average LOS for all patients was 1.9 days versus 3.2 projected days based on APSA guidelines (*P* < 0.0001). Actual versus projected LOS for Grades III to V was 2.5 versus 4.3 days (*P* < 0.0001), and all patients returned to full activity without complication. The use of hemodynamic status to guide management has also been prospectively validated by St Peter et al. [19] and has been subsequently validated in long-term studies by the same group [20].

Before the APSA Trauma Committee guidelines [25], ICU admission was used for patients with AAST Grade IV or higher injuries. However, validation studies showed that admission to ICU based on the grade of injury results in excessive admission of stable patients requiring no intervention [73]. Some data on ICU use based on hemodynamic status rather than grade currently exist. However, Grade V injuries remain independently associated with a high risk of failing NOM and, according to Notrica et al. [22], may still require ICU admission independently from hemodynamic status. A large multi-institutional retrospective review of more than 1800 pediatric solid organ injuries (liver, spleen, kidney, and pancreas) found that NOM fails in very few liver/spleen patients. Among more than 1000 blunt injuries in the series, NOM failed in only 19 patients resulting in shock or bleeding. The median time to operate for all patients was 3 h, with 87% failing by 24 h; thus, only 0.6% of the total number of patients failed after 24 h for all causes [74]. Another series demonstrated only one delayed splenic bleed among 300 patients, and the bleed occurred outside of the period of hospitalization suggested by APSA guidelines [75].

For children, patients and parents, psychological involvement after trauma can be related to abdominal pain. For this reason, family and patient education post-discharge might be considered to reduce the readmission rate [76]. According to Notrica et al. and the ATOMAC guidelines [22], routine re-imaging in all children with blunt splenic or liver injury is not indicated during the observation period.

**Research question 4:** What kind of hemodynamic monitoring is indicated during NOM for splenic trauma according to the injury grade? (***Medium priority***).

**Statement:** No studies focused on hemodynamic monitoring in patients with splenic injuries treated with NOM. No comparative studies focused on the proper monitoring according to the grade of the injury. Consequently, the evidence available is derived from case series and studies designed for other purposes, making the quality of evidence very low. Hemodynamic monitoring in patients with high-grade injuries is performed with continuous monitoring of vital parameters (pulse pressure, cardiac frequency, and peripheral O2 saturation), frequent medical and nursing monitoring and frequent evaluation of serum hemoglobin and hematocrit **[Quality of Evidence: Very low].**

**Recommendation:** There is not enough evidence to recommend specific hemodynamic monitoring in patients with splenic injuries treated with NOM. The panel suggests that all patients treated with NOM for high-grade splenic injuries (WSES Classes II–III, AAST Grade ≥ III) might receive continuous hemodynamic monitoring of vital parameters (pulse pressure, cardiac frequency, and peripheral O_2_ saturation) and frequent serum hemoglobin and hematocrit levels evaluation (every 8 h) **[Strength of Recommendation: Conditional recommendation, based on Very low quality of evidence, Agreement 93.5%]**. In patients with low-grade splenic injuries and stable hemodynamic status (WSES Class I, AAST Grades I–II) treated with NOM, the panel suggests close medical and nursing monitoring with evaluations of hemoglobin and hematocrit levels every 12–24 h if no complication occurs **[Strength of Recommendation: Conditional recommendation, based on Very low quality of evidence, Agreement 91.3%]**.

There are no studies available in the literature focusing on the best hemodynamic monitoring technique to be adopted in patients with splenic injuries treated with NOM; all the available evidence is derived from the description of NOM protocols in studies designed for other purposes; therefore, the quality of the evidence available is overall poor. Most patients with minor injuries (AAST Grade I or II) are admitted to the ward without continuous monitoring. On the other hand, subjects with an injury Grade of III or more receive continuous surveillance of vital signs through continuous monitoring in HDU or ICU.

A survey promoted by the AAST in 2004 showed that there were protocols for NOM of splenic injuries only in 30% of the respondent centers; all the patients with an AAST Grade III or higher splenic injury underwent continuous monitoring [77]. Brillantino et al. in a retrospective study of 87 patients treated with NOM for splenic injuries, described their NOM protocol: each patient was evaluated with repeated arterial gas analysis every 12 h and complete blood cell count every 6 h until stability was reached. There was no mention of any hemodynamic monitoring and the admission to ICU [78]. Teichman et al. described their protocol in a retrospective study including 107 patients: patients with low-grade injuries were on bed rest until two successive serum hemoglobin levels took every 12 h were within 10% of each other. There was no mention of other hemodynamic monitoring. Patients with high-grade injuries were admitted to the ICU until three successive hemoglobin levels, taken every 8 h after the first, were within 10% of each other [17].

In a prospective study of 249 pediatric patients with NOM for solid organ injuries (including also liver trauma), St Peter et al. described their protocol based on the continuous cardiac frequency and pulse oximetry monitoring and repeated evaluation of hemoglobin levels every 4 h until stable [20]. NOM was demonstrated to be feasible also in patients with altered consciousness: Teuben et al. reported their retrospective experience with NOM in 20 patients with GCS < 14. All the patients were admitted to a monitored intermediate care unit or ICU and underwent frequent examinations of vital signs and physical examinations (including hemoglobin level measurements) [79]. Recently, Kumar et al. published a randomized trial where patients treated with NOM for blunt spleen and liver trauma were randomized to early (3 days) or late (5 days) discharge. All patients were treated according to the NOM protocol that included continuous monitoring of vital parameters and urinary output, repeated physical examinations every 6 h, and laboratory investigations [69].

No comparative studies among different hemodynamic monitoring protocols are available in the literature, and no evidence-based conclusions can be made; recommendations made by the panelists are derived from low-quality studies and personal expertise. Therefore, panelists advocate for dedicated studies to evaluate the best hemodynamic monitoring.

**Research Question 5:** When is angioembolization indicated for patients treated with NOM for splenic trauma? (***Urgent priority***).

**Statement:** In stable patients with high-grade splenic injuries (WSES Classes II–III, AAST Grade ≥ III), splenic artery angioembolization (SAE) represents an effective adjunctive tool to NOM, reducing the failure of the conservative approach and the need for surgery. Angiography and SAE should be performed at an early stage when contrast extravasation or vascular injuries (pseudoaneurysm, arteriovenous fistula formation, vessel truncation) are detected on admission CT scan, as well as in high-grade splenic injuries (WSES Class III, AAST Grade IV–V) even if contrast extravasation is not detected. However, small pseudoaneurysms can be safely observed without SAE [**Quality of Evidence: High**]. Some discrepancies in the management of WSES Class II (AAST Grade III) blunt splenic injury without contrast extravasation still exist among the experts. However, based on the available evidence, SAE in WSES Class II (AAST Grade III) splenic injury without vascular extravasation cannot be currently recommended as routine practice. In children, SAE has not been shown to be efficacious [**Quality of Evidence: Low**]. The available literature is inconclusive regarding whether proximal or distal embolization should be used to avoid significant re-bleeding, and larger prospective cohort studies are required. However, both techniques have an equivalent rate of post-procedural splenic infarctions and infections. Minor complications occur more often after distal embolization. This is primarily explained by the higher rate of segmental infarctions after distal embolization [**Quality of Evidence: Low**].

**Recommendation:** The panel suggests splenic artery angioembolization (SAE) as the first-line intervention in patients with hemodynamic stability and arterial blush on CT scan, irrespective of injury grade, where the expertise and resources required to carry out the procedure are readily available **[Strength of Recommendation: Conditional recommendation, based on Moderate quality of evidence, Agreement 93.5%]**. A low threshold for SAE is suggested for patients with WSES Class II (AAST Grade III) blunt splenic injury without contrast extravasation in the presence of risk factors for NOM failure (i.e., age above 55 years old, high injury severity score, the need for red cell transfusions in ED or during the first 24 h, patients on anticoagulant therapy, HIV disease, cirrhosis, and drug addiction) **[Strength of Recommendation: Conditional recommendation, based on Low quality of evidence, Agreement 89.1%]**. The panel suggests angiography and eventual SAE in all hemodynamically stable adult patients with WSES Class III (AAST Grade IV–V) splenic injuries, even in the absence of CT blush, in centers with adequate experience and where SAE is rapidly available, especially when concomitant surgery that requires change of position and that may cause dislodgement of clots and rebleeding (i.e., spinal surgery in the prone position) is needed **[Strength of Recommendation: Conditional recommendation, based on Moderate quality of evidence, Agreement 83.7%]**. The panel suggests SAE be reserved for children who demonstrate evidence of ongoing bleeding with a vascular blush seen on CT **[Strength of Recommendation: Conditional recommendation, based on Low quality of evidence]**. The panel suggests preferring proximal SAE over distal SAE when splenic artery angioembolization is needed **[Strength of Recommendation: Conditional recommendation, based on Low quality of evidence, Agreement 84.8%]**.

Our review of the literature, including systematic reviews, meta-analyses, consensus papers, and retrospective cohort studies [6, 10, 78, 80–89], identified specific indications for angiography and splenic artery embolization (SAE), including vascular injures (contrast extravasation, pseudo-aneurysm, arteriovenous fistula formation, vessel truncation), high-grade (AAST IV–V) splenic injury, with no clear consensus about mandatory SAE in Grade III, large hemoperitoneum, and decreasing hemoglobin. Prophylactic SAE in high-grade splenic injuries, independently from the presence of active bleeding or vascular injuries, has been widely associated with a decrease in NOM failure and a lower incidence of splenectomy, with a splenic salvage rate of over 90% in all injury grades, and greater than 80% for the most severe injuries. In general, proximal SAE has been the preferred technique because the reduction in intra-parenchymal blood pressure prevents late vascular injuries from the onset. Despite this, SAE in splenic trauma still lacks clear indications with various unselected approaches to its use [6].

Arvieux et al. [86] randomized 140 patients with splenic trauma at high risk of rupture (AAST Grade III with large pelvic hemoperitoneum and/or serious damage with a New Injury Severity Score of 15 or more, Grade IV splenic trauma, or Grade V splenic trauma with persisting vascularization of the spleen) to prophylactic SAE (pSAE) or surveillance and then embolization only if necessary (SURV); 133 patients were retained in the study. For the primary endpoint (1-month spleen salvage), data from 117 patients (57 who underwent pSAE and 60 who underwent SURV) were analyzed. The number of patients with at least a 50% viable spleen detected on a CT scan at month one was not significantly different between the pSAE and SURV groups, but, by the day five visit, there were significantly fewer splenic PSAs among patients in the pSAE group than in the SURV group (1.5% vs. 12.3%, *P* = 0.03), significantly fewer secondary embolizations among patients in the pSAE group than in the SURV group (1.5% vs. 29.2%, *P* < 0.001), and no difference in the overall complication rate between the two groups. Between the day five and month one visits, the overall complication rate was not significantly different between the pSAE and SURV groups, and the median length of hospitalization was significantly shorter for patients in the pSAE group than for those in the SURV group (9 days vs. 13 days, *P* = 0.002).

Two different meta-analyses reported consistent results on the effects of SAE on Grades IV–V. Crichton et al. [6] analyzed 23 studies (6684 patients) that compared outcomes in adult blunt splenic injury patients treated with SAE or NOM alone. For Grades I to V combined, there was no difference in the NOM failure rate (SAE 8.6% vs. NOM 7.7%, RR 1.09), mortality (SAE 4.8% vs. NOM 5.8%, RR 0.82), length of hospitalization (11.3 vs. 9.5 days), or blood transfusion requirements (1.8 vs. 1.7 units) between patients treated with SAE and those treated with NOM alone. However, morbidity was significantly higher in patients treated with SAE (SAE 38.1% vs. NOM 18.6%, RR 1.83). Notably, when stratified by grade of splenic injury, SAE significantly reduced the failure rate of NOM in patients with Grade IV and Grade V splenic injuries but had minimal effect in those with Grade I to Grade III injuries. Similarly, Requarth et al. [90] showed that SAE was associated with significantly higher splenic salvage rates in AAST splenic injury Grades IV and V. In this meta-analysis, the failure rate of NOM without SAE was statistically higher than the failure rate of SAE in high-grade splenic injuries (AAST IV–V): 43.7% (95% CI 25.5–63.8) versus 17.3% (95% CI 7.8–34.1) for Grade IV and 83.1% (95% CI 45.2–96.7) verus 25.0% (95% CI 8.7–53.8) for Grade V injuries, respectively.

Bhullar et al. [82] analyzed 539 hemodynamically stable patients with blunt splenic injuries who underwent NOM, of which 104 (19%) underwent SAE, and 435 (81%) were observed without SAE. There was no statistically significant difference in failure of NOM in patients who did not undergo SAE versus those who did for Grades I to III. However, a significant decrease in failure of NOM was noted with the addition of SAE for Grades IV to V: Grade IV (23% vs. 3%, *P* = 0.04) and Grade V (63% vs. 9%, *P* = 0.03).

The absence of blush on CT in low-grade injuries reliably excludes bleeding. Conversely, in high-grade injuries, CT is not accurate enough in excluding vascular injuries. Haan et al. [85] described that 23% of patients with Grade III to V injuries who were embolized after a positive diagnostic angiography had no signs of vascular injury on admission CT scan. Furthermore, Miller et al. [8] demonstrated that after negative angiography, patients were at risk of delayed bleeding, with a failure rate higher than 10% in high grades. The authors suggested that a protocol requiring angiography and SAE for all high-grade spleen injuries slated for NOM leads to a significantly decreased failure rate [89].

The 2020 World Society of Emergency Surgery (WSES) consensus practice on Grade III blunt splenic injury without contrast extravasation [84] found that, among the experts, three factors generated discrepancy in opinion on managing this pattern of injury: the patients' injury severity, the presence of a bleeding diathesis, and associated intra-abdominal solid organs injury. In keeping with the results of the consensus, a low threshold for SAE is suggested for patients with high injury severity, the presence of a bleeding diathesis, and associated intra-abdominal solid organs injury in AAST Grade III blunt splenic trauma without contrast extravasation.

Between 2.3 and 47% CT-detected contrast blush could not be confirmed at the subsequent angiography. In the retrospective study by Alarheyem et al. [91], a total of 143 patients with blunt splenic injuries were found to have CT blush and underwent catheter angiography. However, 24 (17%) showed no evidence of blush on angiography. Patients with CT-angiographic discrepancy were more than twice as likely to re-bleed compared with those with angiographic evidence of blush (25% vs. 10%) in this study, and, according to the authors, this was because, although all patients with a blush on angiography underwent embolization, only 7 of 22 of those with no evidence of blush were embolized. Finally, the authors concluded that in clinical situations where a CT blush is noted secondary to blunt trauma to the spleen, a negative angiogram still carries a significant risk of recurrent hemorrhage. Reason for which consideration for empiric embolization at the time of the initial procedure, even in the absence of blush on angiographic evaluation, is warranted.

Embolization can be completed either by occluding the main splenic artery, referred to as proximal embolization, or by selectively targeting splenic artery branches with visualized injuries on angiography, referred to as distal embolization. Proposed benefits to proximal embolization include speed and ease of procedure, lower cost, and fewer splenic abscesses and infarctions [92–94]. However, the available literature is inconclusive regarding whether proximal or distal embolization should be used to avoid significant re-bleeding, and larger prospective cohort studies are required. In the observational study by Killeen et al. [92], splenic infarcts occurred in 63% of patients after proximal and 100% after distal SAE. Moreover, infarcts after distal embolization tend to be larger, whereas infarcts after proximal embolization tend to be smaller, multiple, and located in the periphery. Although most infarcts resolved without sequelae, seven patients developed gas within an infarct or subcapsular fluid collection. Two collections were drained and found to be sterile, and one patient had a splenic abscess at laparotomy. In the retrospective cohort study by Brahmbhatt et al. [95], sixty patients underwent angiography for splenic injury after blunt traumatic injury, and forty-four patients were embolized. Seventeen patients underwent proximal SAE, and 23 underwent distal SAE. Four patients had a combination of proximal and distal SAE. Eleven patients had subsequent major complications requiring splenectomy. There was no significant difference in major complication rate when comparing proximal SAE 29.4% versus distal SAE 21.7%. However, there was a statistically significant difference in fluoroscopy time between the proximal (10.1 ± 4.2 min) and distal groups (17.8 ± 8.7 min). Other studies [96, 97] showed equivalent results between the two techniques regarding technical and clinical success. Proximal SAE was associated with fewer and smaller infarcts than distal SAE in a series of CT scans of the embolized spleen in another study [98]. Schnüriger et al. [99], through a systematic review and meta-analysis, evaluated the outcomes of proximal versus distal SAE after trauma in a total of 479 embolized patients. The overall failure rate of angioembolization was 10.2%. Re-bleeding was the most common reason for failure, and it did not differ statistically between the used techniques. Conversely, minor complications occurred, especially segmental infarction, statistically and clinically more often after distal than proximal embolization.

Regarding the preservation of splenic immune function, Foley et al. [100] provided low-grade evidence that SAE may reduce immunological complications of spleen trauma and suggest that distal SAE may maintain better function. Other studies evaluating the effects of proximal versus distal SAE on immune function have not shown a difference between these methodologies. However, these studies have included only small data sets and may not have sufficient power to detect any variability [101–103].

**Research Question 6:** Is there a need for radiological follow-up during the hospital stay for patients treated with NOM for splenic trauma according to the injury grade? (***Urgent priority***).

**Statement.** A selective re-imaging strategy appears safe, as re-imaging asymptomatic patients rarely results in intervention. Although limited due to the lack of high-quality research, a follow-up CT scan seems to be justified only in patients with WSES Class II (AAST Grade III) or higher splenic injuries to identify vascular abnormalities after splenic trauma. Indeed, 75–90% of adults with pseudoaneurysm or arteriovenous fistula and all such lesions in the pediatric population occur in patients with injuries of such grade [**Quality of Evidence: Low**]. In higher grade injuries (WSES Classes II–III, AAST Grade III–V), contrast-enhanced ultrasound (CEUS)/CT scan follow-up might be performed in the first 48–72 h to exclude the development of vascular complications [**Quality of Evidence: Low**]. In literature, most delayed splenic complications are diagnosed in the first 7 days from trauma and rarely in AAST Grade I splenic injuries [**Quality of Evidence: Low**].

**Recommendation.** The panel suggests radiological follow-up to be based on clinical findings in WSES Class I (AAST Grades I–II) splenic trauma treated with NOM and suggests against routine imaging follow-up in these patients **[Strength of Recommendations: Conditional recommendation based on Low quality of evidence, Agreement 91.3%]**. The panel suggests repeating imaging with contrast-enhanced ultrasound (CEUS)/CT scan in 48–72 h post-admission and, eventually, at 5–7 days of trauma (only if remarkable changes in CT scan at 72 h are detected, or new signs/symptoms related to the trauma occur) in adult patients with WSES Class II splenic injuries (AAST Grade III) or higher treated with NOM, regardless of whether SAE has been performed or not **[Strength of Recommendation: Conditional recommendation, based on Low quality of evidence, Agreement 82.6%]**.

The role of follow-up imaging and its timing according to the injury grade in patients treated with NOM for splenic trauma is still controversial. As reported in several studies, follow-up imaging with a CT scan might not be mandatory in the absence of clinical signs and symptoms in lower grades (AAST I–II) blunt splenic injuries. The panel conditionally recommends that the best management is to identify people at greater risk of complications and require further treatments or changes in management. According to the 2017 WSES guidelines [1], CT scan repetition during admission should be considered in patients with moderate and severe lesions (AAST III–V or I–V if patients are unstable) or in patients with decreasing hematocrit, vascular anomalies, coagulopathy, underlying splenic pathology and neurologically impaired patients. Follow-up CT scan to report splenic recovery has been regularly challenged in the literature, as it is considered an expensive habit that rarely impacts management decisions. To date, there are neither validated guidelines nor formal consensus about the benefits, methods, and timing of imaging follow-up for splenic trauma.

Routine follow-up imaging in these patients would aim to identify the formation of delayed vascular abnormalities, especially PSAs, and to promptly intervene to avoid NOM failure. However, several authors do not recommend routine follow-up imaging. Liechti et al. [68] evaluated the relevance of follow-up imaging in a retrospective cohort of adult patients with blunt splenic injuries admitted to a Level I trauma center. One-hundred-and-two patients were treated by NOM, with a failure rate of 3.9%. Failure was significantly associated with active bleeding and liver cirrhosis. Eighty patients (78.4%) received follow-up imaging by ultrasound (US) or CT scan. In most cases, routine imaging examinations were conducted without prior clinical deterioration, and 96.4% of these imaging results revealed no new significant findings. Conversely, every failure of NOM was detected following a clinical deterioration in the first 48 h.

Similarly, Thaemert et al. [104] stated that routine follow-up abdominal CT scan in patients with splenic injuries managed with NOM is not required in the absence of clinical indications. Their study obtained 62 follow-up abdominal CT scans in 49 patients with splenic trauma treated with NOM. Information that affected management was evident on only one follow-up CT scan performed in the absence of clinical indications. Also, Sharma et al. [105] and Shapiro et al. [106] showed a limited role of repeat CT scans in NOM of spleen trauma. Shapiro et al., in particular, reported that follow-up CT scans underestimated injury, possibly related to a progression of bleeding and delaying the timing of the operation. Boukar et al. [107] reported evidence suggesting that routine CT follow-up of spleen injuries does not provide significant changes in clinical management. In their systematic review, seven studies reported whether a repeat CT scan during follow-up was routine (74%) or prompted by clinical indication (26%). Notably, only 4% of routine CT scans led to a change in clinical management, whereas, of the repeated CT prompted by clinical indication, 47% led to a change in management.

The presence of PSAs on CT correlates with failure of NOM. Weinberg et al. [108] suggested a systematic approach with follow-up CT scans obtained between 24 and 48 h after admission to detect splenic PSAs as early as possible. In their study, follow-up CT scans at 24–48 h were performed in 269 patients according to protocol. Serial CT imaging resulted in the angiographic detection of 14 (4%) early PSAs and 11 (3%) late PSAs. In this study, PSAs were associated with increasing grades of injury, but almost one-quarter (24%) of PSAs were observed in Grade I and II patients. Embolization was successful in 93% of patients with early PSAs and 91% with latent PSAs. The splenic salvage rate for all patients selected for NOM during the study period was 97%.

Most institutions (62%) involved in an international survey did not report an imaging protocol or follow-up for blunt splenic trauma patients treated with NOM, with 42% of the centers choosing the type and timing of follow-up based on the referring clinician's decision. Among 37% of these institutions, imaging follow-up was indicated in all patients with AAST Grade II or higher splenic injuries, with only 21% performing imaging follow-up in all grades splenic injuries [109]. Similarly, a survey of the Eastern Association for the Surgery of Trauma (EAST) Member Practices in blunt splenic injury reported that only 14.5% of the interviewed would systematically obtain abdominal CT scans in all grades of splenic trauma. Among the rest of the sample, 61.3% would repeat the CT scan if evidence of clinical deterioration, contrast blushing on initial admitting CT scan (37%), and presence of high-grade (IV and V) splenic injury (20%) are present [77].

Some authors suggested considering a follow-up CT only in patients having Grade III or greater splenic injury, as 75–90% of adults with PSA or arteriovenous fistula, and all such lesions in the pediatric population, occur in patients with such graded injury [110]. Weinberg et al. [108] focused on the need to identify early complications such as latent PSA, to avoid failure of NOM. To do this, they conducted a retrospective study in which 341 patients diagnosed with CT abdomen-pelvis at admission underwent NOM for blunt splenic trauma and a follow-up CT scan of the abdomen 24–48 h after injury. In 6% of the patients, early PSA was suspected at admission CT scan, and in 14 of 21 was confirmed at angiography. Follow-up CT scan was performed at 24–48 h after trauma in 269 patients according to the protocol and permitted to diagnose 18 (5%) latent splenic PSA. With regards to the timing of imaging follow-up, we found variability in the literature, with authors suggesting performing a control CT scan between day five and day 30 [86], between 24 and 72 h after admission [88, 89, 108, 109], or within 24 h for CT scan to 10 days post-admission for US [68]. Raza et al. [111] agreed on a selective re-imaging strategy with CT scan only in case of hemoglobin dropping despite three units of blood transfusion, progressive distension of the abdomen, signs of infection, vomiting, hematuria, and tachypnea. With the implementation of these indications, NOM was successful in 963 (89.91%) out of 1071 patients. In their retrospective study, Furlan et al. [112] showed a 23% incidence of delayed splenic vascular injury with follow-up CT performed at a median of 2 days after injury.

In a Japanese study involving 104 patients, delayed formation of PSA was identified with CT scan in 15% of all patients during hospital days 1–8. The authors suggested that follow-up CT should be performed around 1 week after splenic trauma to detect delayed PSA formation [113]. Several studies showed that more than 74% of splenic PSA are detected on follow-up imaging performed 24–72 h after trauma [105, 106, 114, 115]. In 2018 Cimbanassi et al. [88] published the results of an international consensus conference reporting no clear recommendation for follow-up imaging in blunt splenic injury but suggesting, based on experts' opinion, to repeat imaging in 48–72 h in all high-risk patients for late vascular injuries (AAST Grades III–V). Experts previously agreed that follow-up imaging in AAST Grades III to V was appropriate, whilst a consensus was not reached for grade II. No definitive data exist regarding complications rate and short and long-term follow-up strategies for pediatric splenic trauma patients undergoing NOM. Notrica et al. [116] reported, in their prospective study, conducted on 509 pediatric patients with blunt abdominal trauma, a 0.2% incidence of delayed splenic bleeding. Huebner et al. [117] conducted a literature review concerning the role of follow-up imaging of blunt splenic trauma in children. Results were extracted from 26 cohort studies on an overall population of 929 patients who underwent routine follow-up imaging with US, CT, or scintigraphy. No deaths related to splenic injuries were reported, and the incidence of delayed splenic rupture was 0.3% in the general cohort. According to the authors, these findings do not support the use of routine follow-up imaging of children with blunt splenic trauma.

Complications of solid organ injuries treated with NOM requiring intervention are infrequent. In their study, Gates et al. found that re-imaging after 14 days did not lead to intervention in any of the 534 patients who underwent NOM. They proposed a selective re-imaging strategy as a safe and cost-effective strategy. The systematic review by Gates et al. [71] observed that most studies showed a low rate of complications in pediatric patients with post-traumatic splenic injuries, particularly low-grade ones. So, follow-up imaging cannot be considered mandatory for low-grade and uncomplicated injuries or asymptomatic patients, but it may be considered in high-grade injuries for the risk of developing life-threatening complications. Safevi et al. [118] showed a 17% incidence of PSA in AAST Grade IV patients in a population of 186 pediatric patients treated with NOM for isolated splenic or post-traumatic liver injuries. In this retrospective study, all injuries were detected at admission using CT, while follow-up imaging was performed using Doppler US 5 or 7 days after trauma. The results showed two children requiring SAE and one an emergency splenectomy. Only one patient was symptomatic. So, the authors concluded that high-grade splenic and liver injuries are at higher risk of developing complications such as PSA. Thus, they should be subjected to routine follow-up before discharging.

**Research Question 7:** What is the best imaging method for follow-up patients treated with NOM for high-grade splenic trauma in the acute phase during NOM (Ultrasound without contrast, Contrast-enhanced ultrasound, CT-Angio)? (***High priority***).

**Statement:** Although contrast-enhanced CT scan is the gold standard modality for imaging abdominal organ traumatic injury, contrast-enhanced ultrasound (CEUS) has developed a role in the follow-up of traumatic injuries, including splenic trauma. CEUS is a valuable tool for detecting post-traumatic lesions; it is comparable to CT after splenic embolization and may replace CT in follow-up studies in expert hands [**Quality of Evidence: Low**]. CEUS may be considered in children, although repeat imaging in children is rarely needed [**Quality of Evidence: Low**].

**Recommendation:** The panel suggests, in expert hands and dedicated institutions, using CEUS as an alternative imaging modality in the follow-up of conservatively managed splenic trauma to reduce the number of CT examinations, especially in children [**Strength of Recommendation: Conditional recommendation, based on Low quality of evidence, Agreement 91.3%**].

CEUS has been demonstrated to have comparable sensitivity to contrast-enhanced CT scan in the diagnosis of traumatic injuries in patients with low-energy isolated abdominal trauma, with levels of sensitivity and specificity up to 95% [119]. Dormagen et al. [120] reported CEUS was comparable to CT scan in detecting post-traumatic splenic lesions during follow-up. The authors described an overall sensitivity and specificity for all lesions of 87% and 88% at early follow-up and 85% and 95% at late follow-up, respectively. Furthermore, they reported a sensitivity and specificity at early follow-up of 85% and 70% for perisplenic fluid, 80% and 94% for subcapsular hematoma, 83% and 73% for lacerations, and 75% and 87% for infarctions. At late follow-up, the sensitivity and specificity were 60% and 100% for subcapsular hematoma, 91% and 67% for intrasplenic hematoma, 100% and 93% for lacerations, 89% and 100% for scars. Current recommendations provided by the Eastern Association for the Surgery of Trauma (EAST) and the World Society of Emergency Surgery (WSES) [1, 121] identify contrast-enhanced CT scan of the abdomen as the gold standard in the diagnosis of blunt splenic injuries. The use of CEUS as an in-hospital follow-up in blunt splenic injuries is increasing worldwide. Evidence supports the role of CEUS as a real-life, non-invasive, bedside, radiation-free technique that has been proven to be a valid alternative to CT scanning in monitoring traumatic lesions in solid abdominal organs [122–124]. Limitations of the CEUS include lack of 3D scanning and whole-body exploration, difficulties in identifying bowel and mesenteric injuries, and operator dependence [125]. Its use in follow-up would decrease the number of CT scans, particularly where avoiding ionizing radiation is of significant importance, including children and pregnant women or patients with more risk of side effects from iodinated contrast [126]. Valentino et al. [127] studied 133 hemodynamically stable patients with blunt abdominal trauma assessed by US, CEUS, and CT scans. US sensitivity, specificity, and positive and negative predictive values were 70.2%, 59.2%, 74.7%, and 53.7%, respectively, whereas those of CEUS were 96.4%, 98%, 98.8%, and 94.1%, respectively. Similarly, Catalano et al. [128] reported that the extent of 17 splenic injuries on CEUS was comparable with that on CT in 13 and underestimated in 4. In their study, CEUS was shown to be more sensitive than unenhanced US in detecting splenic injury, with a more significant correlation with CT. Tagliati et al. [129] analyzed 139 trauma patients where CEUS and contrast-enhanced CT follow-up were performed, showing diagnostic comparability of 98.6%. Several authors published encouraging results on the use of CEUS in mild-to-moderate blunt abdominal trauma, reporting accuracy comparable to CT in detecting lacerations on the parenchyma of the abdominal organs [119, 120, 122, 126, 128–131], as well as a follow-up after embolization of splenic trauma [120]. Dormagen et al. [120] compared the diagnostic performance of CEUS to CT in trauma patients after SAE. The sensitivity and specificity for CEUS at early follow-up were 85% and 70% for perisplenic fluid, 80% and 94% for subcapsular hematomas, 83% and 73% for lacerations and 75% and 87% for infarctions, respectively. The sensitivity and specificity at late follow-up were 60% and 100% for subcapsular hematomas, 91% and 67% for intrasplenic hematomas, 100% and 93% for lacerations and 89% and 100% for scars, respectively. The overall sensitivity and specificity for all lesions were 87% and 88% at early follow-up and 85% and 95% at late follow-up, respectively. Compared to CT, CEUS underestimated the injury grade in 2/40 cases and overestimated the injury grade in 3/40 cases.

Di Renzo et al. [132] retrospectively studied 46 pediatric patients with isolated and combined post-traumatic injuries for 30 splenic, 15 hepatic and 12 renal injuries. At time zero, 42 patients underwent CT, and only 4 underwent CEUS. During follow-up, imaging was performed in 16 patients within 72 h, in 24 patients between 5 and 10 days post-admission, and in 21 patients between 20 and 76 days post-admission. NOM was successful in all 46 patients; no one needed operative treatment for parenchymal injuries, whereas one patient developed post-traumatic PSA. The authors proposed an imaging follow-up protocol with CEUS in the first 3 days to check early complications, between 5 and 10 days to check the initial healing of parenchymal injuries to allow mobilization, and eventually 1 month after trauma or later to check advanced healing. Durkin et al. [124] sustained that the incidence of PSA is significantly higher in the pediatric literature than previously reported (< 5%) and stated that follow-up imaging with CEUS should be mandatory in all grades of injury for follow-up of hepatic and splenic injuries.

**Research Question 8:** Should patients treated with NOM for splenic trauma (with or without angioembolization) receive vaccinations? (***Medium priority***).

**Statement:** Most patients with splenic injuries treated with NOM, including those treated with splenic artery embolization (SAE), present a significantly lower rate of post-traumatic infections than those who undergo splenectomy [**Quality of Evidence: Moderate**]. SAE does not show higher rates of early and delayed infective complications compared with NOM without SAE [**Quality of Evidence: Low**]. There is insufficient evidence to advise mandatory vaccination in patients treated with NOM for splenic trauma, either with or without SAE [**Quality of Evidence: Moderate**].

**Recommendation:** The panel suggests against routine vaccination for overwhelming post-splenectomy infection (OPSI) from encapsulated bacteria in patients treated with NOM for splenic injury with or without SAE [**Strength of Recommendation: Conditional recommendation, based on Moderate quality of evidence, Agreement 89.1%**]. The panel suggests a tailored approach driven by the immunologic state of the patient before the splenic injury and taking into account possible effects of SAE in losing 50% or more of spleen mass. If 50% or more of the splenic mass is lost, and in every case of WSES Class III (AAST Grade V) injury, patients might be considered as asplenic and potentially more susceptible to OPSI; therefore, they could receive immunization against encapsulated organisms [**Strength of Recommendation: Conditional recommendation, based on Low quality of evidence, Agreement 93.5%**].

The introduction of NOM for the treatment of splenic trauma allowed reducing the number of urgent splenectomies and the impact of the asplenic state on the immunocompetence of these patients, ultimately decreasing the risk of overwhelming post-splenectomy infection (OPSI). The most commonly involved organisms in OPSI are encapsulated bacteria, including Streptococcus Pneumoniae (50% of cases), followed by H. Influenzae and N. Meningitidis. Viruses, fungus and protozoan, are less common [133–135]. In the 2017 WSES guidelines on splenic trauma, adult and pediatric patients undergoing SAE were considered to have less effective immunological function compared to patients treated with NOM without embolization. Despite evidence regarding the possible maintenance of immunologic function, for these patients was considered reasonable to proceed with immunization against encapsulated bacteria as hypo-splenic patients [1]. In current literature, it is still controversial if NOM of splenic injuries, with or without SAE, can be associated with impairment of splenic role in immunocompetence, so it has not been clarified if these patients should be immunized. Some authors argued that immune function might be preserved following SAE [136], and, in 2015, the systematic review by Schimmer et al. [137] concluded that none of the 12 included studies reported an OPSI after SAE. Eleven studies found a preserved splenic function after SAE in adults and children. However, while there is increasing evidence suggesting preservation of splenic immune function following SAE, a universal worldwide standard for quantitative measurement and lack of long-term data makes it difficult to draw definitive conclusions on the subject. There are no specific investigations to evaluate the risk of decreased splenic function in splenic trauma patients undergoing NOM. However, some indirect markers can help to assess the effects of hyposplenism/asplenic state on immunocompetence. Leukocytes, platelets, Howell–Jolly bodies (HJB) and levels of IgA would appear to have increased levels in splenectomized patients, as well as it has been reported a decreased phagocytic activity, serum levels of IgM and opsonin, such as propedin and tuftsi, memory B cell deficiency and impaired T cell-mediated response [138–143]. Skattum et al. [138] conducted a retrospective study to assess long-term splenic functional and immunological outcomes after SAE in children and adolescents. They investigated spleen function after SAE for traumatic injuries on 11 children, performing Doppler US and quantifying blood counts, total immunoglobulins A/M/G, PPV23, H-J bodies, and lymphocytic phenotype levels. None of the patients underwent vaccination after SAE, and no OPSI were reported in the SAE group during the follow-up period (average 4.6 years post-injury, range 1–8 years). All embolized spleens resulted in normal size and were well perfused according to Doppler US. No significant differences were found between SAE and control groups regarding Ig, H–J bodies and PPV23 titles, while all of these appeared to be increased in splenectomized patients. The authors reported that only one of the sierospecific Pneumococcus immunoglobulins (IgM18C) resulted elevated in the SAE patients compared to the control group. Two patients reported an increased incidence of non-invasive infections after SAE. Other studies have investigated splenic function after SAE, most concluding that the spleen's immunocompetence is preserved [138, 142]. In keeping with these findings, several authors supported non-mandatory vaccination after SAE [80]. Olthof et al. [102] performed a prospective study in which the splenic function of patients who underwent SAE was compared to the function of both healthy controls and patients who underwent splenectomy. The median vaccine-specific antibody response of the SAE patients did not differ significantly from that of the healthy controls. In the longitudinal study by Tominaga et al. [141], people with traumatic splenic injury treated at one level II Trauma Center were studied, comparing both SAE and splenectomy patients to a control group of patients with blunt abdominal trauma but no evidence of splenic injury at CT scan. The authors reported that the immunologic state of patients undergoing SAE was similar to controls. Regarding the risk of early infections, Gauer et al. [144] analyzed the outcomes of 155 patients with blunt splenic trauma for 20 years, intending to evaluate the effects of splenic preservation versus splenectomy. Sixty-four patients were treated successfully with NOM, while in the operative group, the overall number of total splenectomies was 57. For the other 34 patients needing surgery, splenic preservation was achieved by splenorrhaphy or partial splenectomy. The spleen was preserved in 98 patients, 64 by NOM (65%) and 34 by operative preservation techniques (35%). Early infections were reported in 3 of 64 patients who underwent NOM (4.7%) and in 5 of 24 patients in the splenic repair group (14.7%). All the infections were proved bacteriologically, and no infections were caused by encapsulated organisms. These results showed a significantly higher rate of early post-traumatic infections in patients who underwent splenectomy than in patients treated with an operative splenic salvage strategy or NOM. There is some evidence that the impact of SAE on the immunologic function of the spleen seems to have no difference whether SAE is performed proximally or distally. As described by Bessoud et al. [83], proximal SAE in blunt splenic injuries is a well-tolerated technique without a major long-term impact on the splenic anatomy and immune function. In this study, all patients (24 of 24) evaluated for exposure-driven immunity against Haemophilus Influenzae B had sufficient immunity. Seventeen of the 18 patients (94%) evaluated for exposure-driven immunity against Pneumococcus had sufficient immunity. Five of the six patients (83%) evaluated for pneumococcus vaccine response had a sufficient response. During the follow-up period, no patient developed severe infections or unexplained fever. Additionally, patients underwent laboratory tests and a Doppler splenic US study to assess SAE's impact on immunological function and anatomy of the spleen. All 24 patients showed evidence of exposure-driven immunity against H. Influenzae; 17 of 18 patients (94%) explored for exposure-driven immunity to S. Pneumoniae polysaccharides had levels of antigen-specific IgG antibodies providing evidence of immunity, as well as 5 of 6 patients (83%) explored for S. Pneumoniae immunity after immunization. Splenic measurements resulted in the normal range after proximal SAE, and the spleen was homogenous in 23 patients (96%). Foley et al. [100] compared the impact of proximal or distal SAE versus splenectomy on splenic immune function as measured by IgM memory B cell levels. They showed a trend toward better preserving IgM memory B cell numbers in patients with distal embolization.

Current Advisory Committee on Immunization Practices (ACIP) recommendations indicate that if 50% or more of the splenic mass is lost, patients should be considered as asplenic and potentially more susceptible to OPSI, and so they should receive immunization against encapsulated organisms [145]. Crooker et al. [146] hypothesized that trauma patients currently receive incorrect vaccination after SAE and that these patients would have poor compliance with ACIP immunization guidelines. The authors argued that the best management strategy for these patients should incorporate a protocol for determining if a patient would be considered asplenic after SAE, and the embolization calculation would be a helpful tool for this.

**Research Question 9:** How long should patients with splenic trauma treated with NOM be followed up after hospital discharge according to the injury grade? (***Medium priority***).

**Statement:** Risk factors for late failure of NOM and hospital readmission include hemoperitoneum with signs of blush at first contrast-enhanced CT and high (WSES Class III, AAST IV–V) grade injuries **[Quality of Evidence: Low]**. There is neither agreement nor sufficient evidence to recommend the optimal length of follow-up after hospital discharge in patients with splenic trauma treated with NOM. The role of imaging has not been cleared out in the post-discharge follow-up **[Quality of Evidence: Very low]**. Imaging follow-up, either by CEUS or CT scan, does not seem to provide clinical benefits in the post-discharge period, and it might be considered in severe injuries (WSES Class III, AAST Grade IV–V) and depending on the level of activity (professional athletes, those practicing high-impact sports, heavy lifting) **[Quality of Evidence: Very low]**. Before returning to major physical activity, imaging follow-up with a contrast-enhanced CT scan or CEUS seems to be indicated to assess the status of the healing process after WSES Class II–III (AAST Grade III–V) splenic injuries treated with NOM **[Quality of Evidence: Low]**. The panel suggests that patients with blunt splenic injuries treated with NOM (with or without SAE) and families be informed of long-term complications and the possibility of NOM failure **[Quality of Evidence: Low]**.

**Recommendation:** The panel suggests selective imaging follow-up at one, three, and (unless imaging confirms healing at 3 months) 6 months after discharge for patients with blunt splenic injuries treated with NOM only in the presence of risk factors for long-term complications and depending on the level of activity (professional athletes, those practicing high-impact sports, heavy lifting). The choice to perform imaging follow-up after discharge includes several considerations, such as the presence of severe splenic injuries (WSES Class III, AAST Grade IV–V); the association with other injuries that would warrant other specific follow-up; the age and expected activity level of the patient post-discharge; the type of NOM utilized (e.g., strictly observational or including interventional radiology); the duration of the hospital stay (with earlier discharge at risk of higher readmission rates) **[Strength of Recommendation: Conditional recommendation, based on Low quality of evidence, Agreement 86.0%]**. The panel suggests performing contrast-enhanced imaging follow-up (CT/CEUS) before returning to major physical activity (2–4 months in high-grade injuries) in adult patients with WSES Class II–III (AAST Grade III–V) splenic injuries treated with NOM **[Strength of Recommendation: Conditional recommendation, based on Low quality of evidence, Agreement 89.1%]**. Pediatric patients could be offered follow-up as outpatient consultation focusing on psychological response to injury and pain management, with further radiological examinations only if clinically indicated **[Strength of Recommendation: Conditional recommendation, based on Very low quality of evidence, Agreement 95.7%]**. The panel suggests imaging follow-up with CEUS before return to major physical activity in children with WSES Class II–III (AAST Grade III–V) splenic injuries treated with NOM **[Strength of Recommendation: Conditional recommendation, based on Low quality of evidence, Agreement 82.6%]**.

Long-term complications of NOM for blunt splenic trauma are delayed splenic rupture, hemorrhage from missed splenic vascular injuries, and development of PSAs. Unfortunately, recent literature that substantiates such a follow-up's duration, timing, or structure is scarce at best, with a void of prospective and endpoint-guided research designs. Hence, the recommendations and outcomes of any suggested surveillance strategy will hinge on some contemporary practice patterns for these injuries. This would include several considerations, such as a single-organ injury or association with other injuries that would warrant other specific follow-ups; the age and expected activity level of the patient post-discharge; the type of NOM utilized (e.g., strictly observational or including interventional radiology); the duration of the hospital stay (with earlier discharge at risk of higher readmission rates).

Risk factors for post-discharge complications and re-admission were retrospectively analyzed by Freitas et al. [147] in a cohort of 2704 patients treated according to NOM principles, both with (257 patients) and without SAE. The thirty-day readmission rate was higher in patients treated with SAE. Primary diagnoses on readmission were spleen injury (36.2%) and respiratory complications (9.05%). Adjunct SAE was reported as an independent risk factor for readmission. Nearly one-fifth of readmitted patients initially managed non-operatively required operative intervention. Patients with splenic injuries treated with NOM and SAE have an increased risk of being re-admitted within 1 year for surgical site infections, pneumonia, urinary tract infections, and sepsis. Crawford et al. [55] carried out a retrospective analysis of 691 patients admitted to a Level 1 trauma center with splenic trauma aimed to identify risk factors for early (within 3 days) and late (after 3 days) NOM failure. Only 7% of patients failed NOM; late failure was observed in 10 (2%) patients. Late bleeding was the cause of NOM failure in all patients with late failure, occurring in 8 ± 6 days after admission. Zarzaur et al. [64] evaluated the risk of re-admission for delayed splenectomy within 180 days from the original injury and reported that it was 1.4% for those treated with NOM and discharged home. The limitations of this study (single-center, retrospective analysis) were addressed by a prospective multicenter study carried out by the same group of authors [67] to assess the risk of delayed hemorrhage after NOM. Among a population of 383 patients, 12 patients underwent in-hospital splenectomy between 24 h and 9 days after injury. Of 366 patients discharged after NOM, 1 (0.27%) required readmission for delayed splenectomy post-injury Day 12. No Grade I injuries underwent delayed splenectomy. The presence of blush signs at the arterial phase of the contrast-enhanced CT at admission was associated with splenectomy. Savage et al. [10] showed that mild injuries had a faster mean time to healing compared with severe (12.5 vs. 37.2 days, *P* < 0.001). Most healings occurred within 2 months, but approximately 20% of each group had not healed after 3 months. Readmission patterns of patients with isolated splenic injuries in the USA showed that around 20% were readmitted within 6 months after discharge (mean time to readmission: 44 days), with only 1.2% needing splenectomy during the readmission after initial NOM [148]. No significant differences were found in readmission rates between the initial chosen management strategy of splenectomy, SAE, or no procedure. A recent series displayed a 2.4% spleen-related 30-day readmission rate after NOM, with the majority (70%) occurring within 7 days of discharge [65]. Several studies have shown that the utilization of SAE serves as an independent risk factor for readmission [65, 150], with SAE showing over double the incidence of infectious complications compared with NOM treatment without SAE [151]. In aggregated data, a recent review stated that routine repeat CT without clinical indication was not helpful in blunt splenic injury managed non-operatively. However, the included studies showed imprecise effect estimates, and some were of low methodological quality [107]. A similar conclusion has been put forward by a single center using the US as a first-line follow-up, stating that clinical findings should indicate the need for radiological follow-up with CT as the preferred imaging modality [68].

In pediatric splenic injury, current guidelines advocate a "less is more" approach to imaging studies with radiation exposure during admission and follow-up [89]. A recent prospective multi-center study looking at pediatric blunt liver or spleen injury concluded that “a selective re-imaging strategy appears safe, and even re-imaging symptomatic patients rarely results in intervention” [116]. This can be seen in conjunction with previous reviews on the matter, which do not support the use of routine follow-up imaging of all children with blunt splenic trauma [117, 150]. Long-term follow-up might play a role in dealing with abdominal pain and psychological repercussions after injury in patients and their relatives [76]. Such indications for outpatient consultations, addressing post-traumatic stress responses after hospital discharge in pediatric and adult patients, are often overlooked and must be studied further.

Healing of blunt pediatric splenic injury seems related to injury severity and the difference in the mean time to healing among all grades is significant. Dickinson et al. [151] conducted a retrospective study between 2000 and 2014 on 214 patients subjected to NOM for blunt splenic injury, of whom 152 (71%) underwent follow-up imaging from 4 to 74 weeks after trauma. The timing of post-discharge imaging was displayed as a ROC curve, and the likelihood of identifying all healed spleens was compared with the likelihood of identifying healing and non-healed spleens for post-injury weeks. The timing to confirm healing of low-grade splenic injuries (AAST Grades I and II) appeared to be optimal around 7–8 weeks post-injury, while no recommendations were possible for high-grade injuries (AAST Grades III–V). In 2018, Tagliati et al. [129] prospectively studied 139 trauma patients with CT-diagnosed spleen injury who underwent NOM. CEUS follow-up was conducted with serial examinations at 1–3–7–15–30–60–90–180 days after trauma until healing of the splenic injury was achieved. CEUS showed 12 delayed splenic vascular injuries and five delayed active extravasation, while CT diagnosed 16 delayed complications in these 17 patients. From these data, CEUS and CT diagnostic comparability was 98.6%. Thirteen patients with delayed complications diagnosed with CEUS underwent angiography that confirmed 12 delayed splenic injuries. CEUS, compared to digital subtraction angiography, showed a positive predictive value of 91.7% and a sensitivity of 100%. In this study, all delayed splenic complications were diagnosed in the first 7 days and none in AAST Grade I splenic injuries. Thus, the authors indicated CEUS could be used in patients with Grade > II injuries to assess delayed complications and to reduce CT examinations, using a pre-established interval of US exams (e.g., 1–3–7 days after trauma). In a retrospective study by Smith et al. [66], the authors included 154 patients aged > 16 years with blunt splenic injury and followed them for at least 30 days after trauma. Of these, 148 underwent CT at admission, while 6 of 154 needed immediate laparotomy and splenectomy for hemodynamic instability. Of patients who underwent CT, ten needed subsequent laparotomy to manage their injuries, 26 underwent angiography with SAE and one only angiography. Fourteen (16.1%) patients bled during NOM in a median of 2.3 days from injury, and eight re-bled after SAE in a median of 2 days. No AAST Grade I injuries and 3.3% Grade II bled during NOM, while higher grade injuries (Grades III–V) were a significant predictor of the failure of NOM with or without SAE (*P* = 0.001, OR 15.6, 95% CI 3.1–78.9) as well as intraparenchymal or subcapsular haematoma (*P* = 0.004, OR 10.9, 95% CI 2.2–55.1). A retrospective analysis carried out at a Level I pediatric trauma center among 222 patients showed that 71% of patients underwent additional imaging 2–74 weeks post-injury before returning to physical activity. The optimal timing for post-injury imaging for Grades I–II was 7–8 weeks, whereas healing of higher-grade injuries could not accurately be predicted [151].

**Research Question 10:** How long should patients treated with NOM for splenic trauma abstain from major physical activities based on the grade of the splenic injury? (***High priority***).

**Statement.** Although there is a lack of high-quality research on the duration and intensity of restricted activity and return to play after blunt spleen injuries treated with NOM, the current trend is toward shorter post-discharge follow-up with earlier return to daily activity. Specifically, physical activity restrictions limited to 4 weeks after the injury, irrespective of injury grade on CT, seem safe in pediatric patients. Non-contact activity, including school, can be allowed safely after discharge [**Quality of Evidence: Low**].

**Recommendation:** The panel suggests that major activity restrictions (athletic activities, no-contact sports, heavy lifting) in adults with splenic trauma treated with NOM might be prescribed for 3–5 weeks in low-grade splenic injuries (WSES Class I, AAST Grades I–II) and up to 2–4 months in high-grade injuries (WSES Classes II–III, AAST Grade III–V). A follow-up imaging with contrast-enhanced CT/CEUS is suggested before return to full activities to confirm healing [**Strength of Recommendation: Conditional recommendation based on Low quality of evidence, Agreement 89.1%**]. The panel suggests that major activity restrictions in children with splenic trauma treated with NOM could be limited to 4 weeks after the injury, irrespective of injury grade on CT scan [**Strength of Recommendation: Conditional recommendation based on Low quality of evidence, Agreement 87%**].

Although there is a lack of evidence-based guidelines regarding the duration and intensity of restricted activity and return to play after blunt spleen injuries treated with NOM, high-risk activities such as biking, skiing, contact sports, and fighting sports are generally restricted for up to 3 months after trauma. However, the current trend is toward an earlier return to activity. This trend is driven by growing evidence that this appears to be a safe practice with decreased healthcare and societal costs. Although late complications of NOM are known to occur, surgeons and researchers believe their occurrence is independent of activity restriction. So, it is unlikely that longer periods of activity restriction will affect their incidence. Activity restriction may be suggested for 4–6 weeks in minor injuries and up to 2–4 months in moderate and severe injuries, according to the WSES guidelines [1]. This suggestion was based on low-quality studies in which the return to regular activity can occur 2.5–3 months after splenic trauma treated with NOM [152, 153]. However, other authors suggested activity restriction of 2 weeks for mild injuries with a return to full activity after 6 weeks and up to 4–6 months for patients with more severe injuries [10, 154].

Given the lack of solid evidence, consensus studies in this research field represent the only available literature. In a Delphi study by the PYTHIA collaborative group [155], recommendations regarding return to full activity varied by the experts' perceived risk to the patient and by injury grade. Agreement regarding recommendations for return to activity was then limited. Especially, a great diversity of opinion concerning the return to contact sports raised. The first Delphi round showed that 3 months of rest was frequently recommended. Zarzaur et al. [154] surveyed activity level recommendations among American Association for the Surgery of Trauma (AAST) members. For a sedentary patient, respondents' recommendations were more conservative as the grade of injury increased (low grade, < 8 weeks; higher grade, varying). For professional football players, the recommendations depended on injury grade (low grade, 4–8 weeks; higher grade, more extended periods or return after showing that the injury was healed on CT scan). For AAST Grade IV or V injury, some of the experts even recommended permanent exclusion from full activity. The period needed before releasing patients back to full activity was a matter of debate also in the Eastern Association for the Surgery of Trauma (EAST) practice management guideline on NOM of blunt splenic injuries [89]. The duration and intensity of restricted activity after discharge remained among the unanswered questions included in the "topics for future investigation." Most respondents (78.1%) relied on clinical judgment alone for activity recommendations in lower grades of injury, while a higher reliance on CT findings was used for AAST Grades IV and V (49.8%). Similarly, Fata et al. [77] found that clinical judgment was the predominant factor cited by EAST members in return-to-activity decisions for all grades of splenic injury. Most EAST members (81%) did not use CT scans following discharge for Grade I and II injury to make activity recommendations. However, the proportion using CT scan increased steadily for higher grades of injury. Concerning follow-up and discharge instructions, 62.9% of the sample chose to allow patients with AAST Grade I and II injuries to return to light housework, office work or low-impact aerobic activity within 2 weeks of their initial trauma. For Grade III injury, 29.6% of respondents chose to allow light activity at 2 weeks. For Grades IV and V, 22.4% of the experts chose to lift light activity restrictions as early as 2 weeks from their injury. Two-thirds of the sample chose to allow patients with Grades III or higher to return to light activities within 4 weeks to 2 months of their injury. For higher injury grades, 10.9% would restrict activities over 3 months. Recommendations concerning strenuous activity (defined as running, lifting over twenty pounds, construction work or manual labor) showed more variation with higher grades of injury. For Grades I and II, the majority of EAST members (74%) chose to restrict strenuous types of activities until 6 weeks. For Grades IV and V, 16.9% of the membership advised strenuous activity confinement for longer than 3 months. For Grade III injuries, 19.8% of the sample would allow the return to full activity within 6 weeks, 56% within 2–3 months and 19.2% within 4–6 months. For Grade IV and V injuries, the majority of respondents were divided among 2–3 months (45.8%) and 4–6 months (31%). With Grade IV and V, 5% would choose to restrict activity for a period longer than 6 months.

Pediatric studies suggested activity restriction limited to 8 weeks [72], or grade of injury plus 2 weeks, in keeping with the APSA guidelines [25]. However, in a 3-year prospective study on 44 patients with blunt liver and spleen injuries published after the APSA guidelines [70], all patients had activity restrictions limited to 4 weeks after the injury, irrespective of injury grade on CT. Non-contact activity, including school, was allowed after discharge. Patients were followed-up at 1 month with ultrasonographic imaging, and no delayed bleeding was detected. This study suggests that the management of pediatric solid organ injuries should be guided by hemodynamic status and not injury grade on CT, and activity restrictions could be shorter than recommended in the APSA guidelines for all injury grades. Similarly, Notrica et al. [156] evaluated activity restriction adherence to the APSA guidelines and found no difference in frequencies of bleeding or emergency department re-evaluation between patients adherent or non-adherent to the APSA activity restriction guideline. Other case series addressed the topic of activity restriction specifically following splenic injury [157–161]. Some of these studies utilized activity restrictions ranging from 4 to 12 weeks. Lynch et al. [162] evaluated radiographic evidence of healing using weekly ultrasound of the solid organ (liver or spleen) to determine when it was safe to return to athletic activity. They found that healing took place between 3 and 21 weeks and correlated with the grade of injury. Another study used CT to assess the healing with a mean time to complete healing of 87 ± 8 days post-injury [163]. However, no correlation between radiographic evidence of healing and clinical parameters can be made to guide the length of activity restriction. According to Gates et al., restricting activity to the grade of injury plus 2 weeks is safe. However, shorter periods need to be investigated prospectively [71].

**Research question 11:** Which is the best treatment of post-angioembolization necrosis of the spleen for patients with splenic trauma treated with NOM? Surgery or radiological drainage? (***High priority***).

**Statement:** Splenic abscess following NOM for splenic injuries is an infrequent complication. In the case of a splenic abscess, surgery and percutaneous drainage seem similar in safety and efficacy, but the quality of supporting evidence is poor **[Quality of Evidence: Low]**.

**Recommendation:** The panel suggests considering percutaneous drainage as the first-line treatment of splenic abscesses after NOM in case of availability of the interventional radiology technique, adequate skills, and technical feasibility **[Strength of Recommendation: Conditional recommendation based on Low quality of evidence, Agreement 95.7%]**. In case of failure of percutaneous drainage (intense and persistent pain in patients with sepsis) or unavailability, the panel suggests performing splenectomy, open or laparoscopic, according to local expertise **[Strength of Recommendation: Conditional recommendation based on very Low quality of evidence, Agreement 97.8%]**.

Splenic abscess and splenic infarction are possible complications of NOM in splenic trauma; the presence of necrotic tissue without vascular supply after trauma or after SAE is recognized as risk factors for developing necrosis and splenic abscesses. A retrospective review of a large population of 17,857 patients treated with NOM for traumatic splenic injury reported a 3% incidence of splenic infarction and 3.9% incidence of splenic abscesses as complications of NOM [65].

Treatment of splenic abscesses is based on the administration of high-dose broad-spectrum antibiotics. Antibiotic therapy is of paramount importance while further therapeutic arrangements are made, and the choice of antibiotics might be guided by the culture results. However, medical treatment alone is not recommended because of medical therapy's low effectiveness in controlling the source of infection. Splenic abscesses could be treated with splenectomy or percutaneous drainage that permits splenic salvage. Splenectomy is preferable in case of multiple or multilocular abscesses, in case of contraindications to percutaneous drainage, or case of patients with no response to medical therapy after percutaneous drainage. It may be performed either laparoscopically or through laparotomy. When feasible, laparoscopic splenectomy is an alternative to the open method. It is associated with faster postoperative recovery and reduced hospital stay [164].

Gutama et al. in 2022 published a systematic review of studies comparing percutaneous drainage and splenectomy for splenic abscesses. They retrieved 46 studies for 589 patients, 288 underwent splenectomy, and 301 underwent percutaneous drainage. The overall mortality rate was similar (12% for splenectomy compared with 8% for percutaneous drainage), and there were no substantial differences in the overall morbidity rate (26% vs. 28%). The two treatments resulted in nonsignificant risk difference for death (− 4% 95% CI − 11% to 3% favoring percutaneous drainage) and for morbidity (− 3% 95% CI − 15% to 10% favoring percutaneous drainage).

The meta-analysis included patients with splenic abscesses of all etiologies, and only a smaller part of the patients were trauma patients; the generalizability of the evidence is limited since most patients had significant comorbidities and were poorly comparable with trauma patients [165].

A proposed alternative for treating splenic abscesses is the EUS-guided transgastric drainage of the abscesses: two case reports reported the technique's feasibility in selected patients. None of the patients was treated for splenic abscess following NOM for splenic traumatic injuries; therefore, evidence is poorly generalizable to trauma patients; more evidence is needed to evaluate the role of this technique in the management of splenic abscess [166, 167].

## Conclusions

This international consensus project aimed to develop evidence-based recommendations regarding the optimal strategies for follow-up of patients with splenic injuries treated with NOM. Evidence-based solid approaches in the follow-up strategies during NOM for splenic trauma are still lacking. However, using a modified Delphi method, a consensus was reached on eleven clinical research questions and 28 recommendations with an agreement rate ≥ 80% among a group of internationally recognized acute care and trauma specialists. This consensus suggested allowing early mobilization within 24 h in adult patients with low-grade splenic injuries treated with NOM. The panel suggests that patients with WSES Class II (AAST Grade III) splenic injuries can be mobilized after 2 days from the trauma if no other contraindications exist. In patients with high-grade splenic injuries, if no other contraindications to early mobilization exist, the patient can be mobilized safely after 2 days from the trauma when three successive hemoglobins 8 h apart after the first hemoglobin check are within 10% of each other and if clinical parameters remain stable. In the absence of specific complications, the panel suggests DVT and VTE prophylaxis with LMWH to be started within 24 h from hospital admission for patients with WSES Class I (AAST Grade I–II) and within 48–72 h for those with WSES Class II–III (AAST Grade III–V) splenic injuries. The panel suggested 1 day (for low-grade splenic injuries) to 3 days (for high-grade splenic injuries) of hospital admission for adult patients, with the duration of stay based on hemodynamic status, hemoglobin and hematocrit stability, and results of the follow-up CEUS/CT scan at 48–72 h. The panel suggests that early discharge after NOM, especially for patients with WSES Class II–III (AAST Grade ≥ 3) injuries, be at least accompanied by an explicit patient and caregiver education regarding the risk of outpatient rupture; in every case, an outpatient clinical follow-up, telephone, GP-follow-up, or community nurse follow-up after 5–7 days was suggested. Where the expertise and resources required to carry out the procedure are readily available, SAE represents the first-line intervention in patients with hemodynamic stability and arterial blush on CT scan, irrespective of injury grade. A low threshold for SAE was suggested for patients with WSES Class II (AAST Grade III) blunt splenic injury without contrast extravasation in the presence of risk factors for NOM failure (i.e., age above 55 years old, high injury severity score, the need for red cell transfusions in ED or during the first 24 h, patients on anticoagulant therapy, HIV disease, cirrhosis, and drug addiction). Moreover, the panel suggested angiography and eventual SAE in all hemodynamically stable adult patients with WSES Class III (AAST Grades IV–V) splenic injuries, even in the absence of CT blush, in centers with adequate experience and where SAE is rapidly available, especially when concomitant surgery that requires a change of position and that may cause dislodgement of clots and rebleeding is needed. Conversely, in children, the panel suggests SAE be reserved for children who demonstrate evidence of ongoing bleeding with a vascular blush seen on CT. Repeating imaging with contrast-enhanced ultrasound (CEUS)/CT scan in 48–72 h post-admission was suggested in adult patients with WSES Class II splenic injuries (AAST Grade III) or higher, regardless of whether SAE has been performed or not. The panel suggested against routine vaccination for OPSI from encapsulated bacteria in patients treated with NOM for splenic injury with or without SAE in favor of a tailored approach that considers the possible effects of losing 50% or more of spleen mass. Finally, for adult patients, the panel suggested major activity restrictions (athletic activities, no-contact sports, heavy lifting) for 3–5 weeks in low-grade splenic injuries (WSES Class I, AAST Grades I–II) and up to 2–4 months in high-grade injuries (WSES Classes II–III, AAST Grades III–V).

This consensus document could help guide future prospective studies aiming at validating the suggested strategies through the implementation of sizeable prospective trauma databases and the subsequent production of internationally endorsed guidelines on the issue. At the same time, a research agenda is necessary on the areas of research which should be investigated in the future: at the moment, the evidence to provide the surgeons with suggestions or recommendations is overall poor. The experts recognized that there is a substantial lack of evidence about the type and timing of hemodynamic monitoring, the choice between proximal and distal SAE, the use of preemptive angiography and SAE in adult trauma patients with WSES Class III splenic injuries, the use of CEUS as an alternative imaging modality to CT, and the duration of major activity restrictions in adults with splenic trauma treated with NOM.

## Data Availability

Not applicable. All data were obtained through focused searches involving MEDLINE (via PubMed), Google Scholar, the Cochrane Central Register of Controlled Trials, and EMBASE online databases.

## Appendix 1: Comments from the experts' panel

***Research Question 1:*** No need for bed rest for Grade I–II splenic injuries in children (**KS**). Scheduled serial hemoglobin measurements are unnecessary, increase cost, and rarely change management, especially in children. The choice to repeat hemoglobin should be based on physiologic signs of ongoing bleeding. The duration of bedrest should be based on clinical findings, not the grade of injury (**DB**). No bed rest is needed just because of the injury. Patients may stay in bed if unwell (**JG**). There is no reason patients need to be at bed rest, unless unwell (**TS**). My practice used to be: Grade = days but agree with the recommendations (**AL**). In patients with high-grade splenic injuries (WSES Classes II–III, AAST Grade III–V), if no other contraindications to early mobilization exist, the patient can be mobilized safely after 48 h (**GF**). It is too early to mobilize AAST Grades III–V adults depending on the hemoglobin level (**FAZ**). Evidence on mobiliztion based on hemoglobin drop is too weak (**IC**).

***Research Question 2:*** For patients with AAST Grade I–II splenic injuries DVT/VTE prophylaxis should be started within 24 h (**EM, KI**). DVT/VTE prophylaxis should be started within 24 h of stable blood count (hematocrit and hemoglobin) (**JG**). Mobilization within 24 h is safe if there is no evidence of bleeding, particularly in low-grade injuries (**WB**). NOM patients can start DVT prophylaxis immediately (**TS**). NOM patients can start DVT prophylaxis 72 h after trauma (**IW**). Subcutaneous LMWH should be administere till being full mobilized (**FAZ**). The uncertainty in the timing should also be considered: 48–72 h seems more appropriate. Again the rationale is that reactionary haemorrhage usually occurs within 48 h (**KG**).

***Research Question 3:*** The length of admission for isolated splenic injury should be based on clinical findings and not injury grade with early discharge if feasible (**DB**). The patient's social status and compliance level, which are key factors in decision-making, should be taken into account (**ZB**). As AAST Grade V injuries still remain independently associated with a high risk of failing NOM, 1 day of in-hospital follow-up for patients with AAST Grade I–II splenic injuries, 2 days for those with AAST Grade III, and 3 days for AAST Grade IV–V is suggested. The patients with high-grade injuries should be discharged with no complaints and stable hemoglobin, and with good explicit patient and caregiver consultation (**ET**). Five days of hospital admission for AAST Grade I–III and 7 days for AAST Grade IV–V injuries. The major concern of reducing the hospital stay to 1 or 3 days depending on the evidence alone has one shortage which is this recommendation is built up on group data. Occasionally a patient may get a rupture and die. If these recommendations are adopted, they should be within clear guidelines which are adopted by hospital administrations for medicolegal issues (**FAZ**). Length of hospital stay should be based on clinical condition, not an abritrary number based on AAST Grade (**IC**). Difficult to abandon AAST Grade plus 1 day in children without clear alternative. If so, the approach based on hemodynamic status should be outlined (**JWL**). In UK, many primary consultations with GP happen over phone. There is also a system of GP nurse/community nurse follow-up for certain conditions in UK. I cannot see any reason why every AAST grade I or II injury should be followed-up in outpatients after a week. A more acceptable statement can be "and in every case, a clinical follow-up at outpatient, telephone, GP-follow-up, or community nurse follow-up after 5–7 days is recommended" (**KG**). The term "monitored setting" could imply a higher level of care such as ICU or intermediate/step-down unit. This is probably not necessary, especially in low-grade injuries (**WB**).

***Research Question 4:*** Scheduled serial hemoglobin measurements are unnecessary in children. One repeat hemoglobin should be obtained prior to discharge (**DB**). Patient with high-grade splenic injuries should be admitted to high dependency units for the continuous monitoring (**OC**). For patients with low-grade injuries, four-hourly heart rate, blood pressure, and clinical conditions monitoring should be enough in the absence of other injuries (**GT**). There should be a time-frame: low-grade injuries may not require monitoring for more than 1 day (**WB**). Hematocrit measurement is better than hemoglobin measurement for monitoring (**IW**). For low-grade injuries and stable haemodynamic status (pulse rate, blood pressure, urinary output), twice daily blood tests seem to be too frequent. Perhaps, change 'low-grade splenic injuries' to 'low-grade splenic injuries and stable hemodynamic status' and change the frequency of evaluation of hemoglobin levels to 12–24 h with more frequent monitoring in people with unstable hemodynamic status (**KG**).

***Research Question 5:*** SAE, when indicated (AAST Grade IV ore more) should be proximal to allow the start of LMWH (**OC**). The indication for SAE must be take into account the need for concomitant surgery( i.e., spinal surgery in the prone position, where change of position may cause dislodgement of clots and rebleeding). In those cases, SAE is indicated also in the absence of active blush (**GT**). Small PSAs can be safely observed without SAE (**TS**). The age of 55 is low to subject a patient for an invasive interventional procedure. I would stick to geriatric age which is 65 (**FAZ**). I disagree that all AAST Grade IV and V injuries should undergo SAE. However, I recommend AAST Grade IV and V injures should undergo CT-Angio or Angiography prior to discharge from the hospital ro rule out PSAa (**RC**). I agree with SAE for all AAST Grade V splenic injuries, but would only do for Grade IV with blush. I also believe preemptive SAE is appropriate for AAST Grade ≥ III if the patient requires anticoagulation (**EM**).

***Research Question 6:*** Routine follow-up imaging is unnecessary in children regardless of injury grade (**DB**). Routine follow-up with CT scan adds expense and radiation exposure to these patients without a real clinical benefit (**JG**). LMWH should be started after follow-up CT/CEUS in AAST Grade III or higher and hospital discharge should be postponed to the follow-up imaging (**OC**). Evidence for benefit of intensive radiological follow-up is too weak (**IC**). All AAST III–V Grades should undergone imaging follow-up to exclude PSAs within the first 2 weeks (**DD**). I do not repeat CT-Angio in 48–72 h routinely. It is too expensive and unnecessary, as the characteristics of the injury do not change substantially in such a short period of time (**RC**).

***Research Question 7:*** Low-dose CT scan as preferred method is an option for imaging follow-up (**AL**). In the choice of the radiological method to follow-up patients with high-grade splenic injury, one must take into account the not-diffuse availability of CEUS and the long-term risk of radiation-associated proliferative disorder with CT scan, in particular in children (**GT**). CT scan with contrast is most widely available; CT angio unnecessary (**WB**). There is still uncertainty around whether CEUS is as good as contrast enhanced CT scan (**KG**).

***Research Question 8:*** Vaccination to all patients with Grade V splenic injuries is my option (**EM**). No data to support the practice. Proximal embolization likely makes the spleen somewhat ischemic but collateral flow is formed. Vaccines are relatively safe so erring on the side of giving vaccines selectively makes sense to me (**TS**).

The threshold to vaccinate after SAE should be a lot lower than 50% loss of parenchyma (**RC**).

***Research Question 9:*** Selective re-imaging may help in people who wish to return to high impact sports, playing competitive football, skydiving etc. (**TS**). With the exception of professional athletes and those practicing sports contact, nobody needs repeat imaging, except for persistent symptoms or complications (**RC**). Patients should have follow-up imaging at 1, 3, and 6 months until healing is confirmed if they have risk factors for delayed/secondary splenic rupture (**EM**).

### ***Research Question 10:*** -

***Research Question 11:*** Another option, in case of unavailability of the interventional radiology technique or lack of adequate skills, can be the referral to another hospital for percutaneous drainage (**FF**). Intense and persistent pain in patients with sepsis may not improve with percutaneous drainage and should undergo splenectomy (**RC**).

## Abbreviations

AAST: America Association for the Surgery of Trauma
ACIP: Advisory Committee on Immunization Practices
APSA: American Pediatric Surgical Association
CEUS: Contrast-enhanced ultrasound scan
CT: Computed tomography
DVT: Deep vein thrombosis
EAST: Eastern Association for the Surgery of Trauma
ED: Emergency Department
ERT: Evidence Review Team
GCS: Glasgow coma scale
HDU: High dependency unit
ICU: Intensive care unit
ISS: Injury severity score
LMWH: Light molecular weight heparin
LOS: Length of hospital stay
NOM: Non-operative management
OPSI: Overwhelming post-splenectomy infection
PE: Pulmonary embolism
PSA: Pseudo-aneurysm
SAE: Splenic artery embolization
UH: Unfractionated heparin
US: Ultrasonography
VTE: Venous thromboembolism
WSES: World Society of Emergency Surgery
WTA: Western Trauma Association
